# Effects of Cortical FoxP1 Knockdowns on Learned Song Preference in Female Zebra Finches

**DOI:** 10.1523/ENEURO.0328-22.2023

**Published:** 2023-03-28

**Authors:** Fabian Heim, Simon E. Fisher, Constance Scharff, Carel ten Cate, Katharina Riebel

**Affiliations:** 1 Institute of Biology, Leiden University, Leiden 2333BE, The Netherlands; 2 Language and Genetics Department, Max Planck Institute for Psycholinguistics, Nijmegen 6525XD, The Netherlands; 3 Institute of Biology, Freie Universität Berlin, Berlin 14195, Germany; 4 Donders Institute for Brain, Cognition and Behaviour, Radboud University, Nijmegen 6525HR, The Netherlands; 5 Leiden Institute for Brain and Cognition, Leiden 2300RC, The Netherlands

**Keywords:** FOXP, songbird, vocal learning

## Abstract

The search for molecular underpinnings of human vocal communication has focused on genes encoding forkhead-box transcription factors, as rare disruptions of FOXP1, FOXP2, and FOXP4 have been linked to disorders involving speech and language deficits. In male songbirds, an animal model for vocal learning, experimentally altered expression levels of these transcription factors impair song production learning. The relative contributions of auditory processing, motor function or auditory-motor integration to the deficits observed after different FoxP manipulations in songbirds are unknown. To examine the potential effects on auditory learning and development, we focused on female zebra finches (*Taeniopygia guttata*) that do not sing but develop song memories, which can be assayed in operant preference tests. We tested whether the relatively high levels of *FoxP1* expression in forebrain areas implicated in female song preference learning are crucial for the development and/or maintenance of this behavior. Juvenile and adult female zebra finches received FoxP1 knockdowns targeted to HVC (proper name) or to the caudomedial mesopallium (CMM). Irrespective of target site and whether the knockdown took place before (juveniles) or after (adults) the sensitive phase for song memorization, all groups preferred their tutor’s song. However, adult females with FoxP1 knockdowns targeted at HVC showed weaker motivation to hear song and weaker song preferences than sham-treated controls, while no such differences were observed after knockdowns in CMM or in juveniles. In summary, FoxP1 knockdowns in the cortical song nucleus HVC were not associated with impaired tutor song memory but reduced motivation to actively request tutor songs.

## Significance Statement

Vocal production learning in humans and birds requires auditory memory formation and recall. Human *FOXP1* mutations are associated with broad neurodevelopmental disorders including speech and language impairments. In juvenile male zebra finches knockdowns of the avian ortholog, FoxP1, in regions relevant for song learning impair song copying. Whether FoxP1 is relevant for auditory learning is unknown. Studies in nonsinging females make it possible to test this. We report that FoxP1 knockdowns in caudomedial mesopallium (CMM) during development and adulthood do not affect auditory learning, while knockdowns in the premotor nucleus HVC in adult but not juvenile female zebra finches reduce tutor song preference and motivation to receive playbacks. These findings support roles of FoxP1 in auditory perception and motivation to hear song.

## Introduction

The discovery of associations between developmental speech and language impairments and rare heterozygous mutations of the Forkhead-box protein P2 (*FOXP2*) gene (Lai et al., 2001) initiated investigations into the neurogenomic basis of vocal learning (for review, see Fisher and Scharff, 2009; Deriziotis and Fisher, 2017; Fisher, 2019). Mutations of *FOXP1*, a paralogue of *FOXP2*, are associated with a multifaceted neurodevelopmental syndrome encompassing phenotypes such as autism spectrum disorder (ASD) and/or cognitive impairments which often affect speech and language (Sollis et al., 2016, 2017). Moreover, heterozygous loss-of-function variants in another paralogue, *FOXP4*, were recently implicated in a developmental disorder characterized by speech/language problems and variable congenital abnormalities (Snijders Blok et al., 2021). The homology between *FoxP* genes, and especially *FoxP1* and *FoxP2*, across vertebrates (Mazet et al., 2003; Hannenhalli and Kaestner, 2009) spurred comparative research on their functions in suitable animal models.

Because sensory and motor learning circuits mediating avian vocal learning are well characterized (Nottebohm et al., 1990; Mooney, 2009; Condro and White, 2014), bird song learning can contribute to the understanding of sensorimotor and auditory processes of human speech acquisition, since parallels between vocal learning in songbirds and humans range from behavioral to molecular features (Doupe and Kuhl, 1999; Jarvis, 2019; Hyland Bruno et al., 2021). This allows for localized knockdown studies of mRNA as a means to identify neuromolecular underpinnings of vocal learning. Knockdowns of FoxP1, FoxP2, and FoxP4 in Area X, a nucleus in the basal ganglia, impair song learning in juvenile male zebra finches (Haesler et al., 2007; Norton et al., 2019). Vocal learning requires song memorization, sensory feedback and motor practice, and brain-expressed FoxP proteins might influence any one or a combination of these underlying mechanisms. Indeed, FoxP1 knockdowns in HVC (acronym used as a proper name) before tutor exposure impair song learning in male juvenile zebra finches (Garcia-Oscos et al., 2021).

Song production is sexually dimorphic as only males sing, but both sexes memorize tutor song as juveniles and as adults prefer to hear the songs they were exposed to early in life (Clayton, 1988; Houx and ten Cate, 1999; Riebel et al., 2002; Riebel, 2003a, 2009). Song preference learning thus provides a unique opportunity to investigate tutor song memorization independent of motor learning (Riebel et al., 2002). *FoxP1* could be involved in this process because it is expressed in HVC and caudomedial mesopallium (CMM) of both sexes (Teramitsu et al., 2004; Chen et al., 2013; Mendoza et al., 2015; this study) although it should be noted that HVC is significantly smaller in females when compared with males (Nottebohm and Arnold, 1976; Nixdorf-Bergweiler, 1996). Both HVC and CMM are involved in song memory, auditory perception and auditory learning (Bell et al., 2015; Roberts et al., 2017; Soyman and Vicario, 2017; Inda et al., 2020, 2021). Other auditory areas which play important roles for song memory, preference and discrimination, such as NCM or NCL, show no elevated FoxP1 expression in comparison to the surrounding tissue in males, while this has not previously been formally evaluated in females (Haesler et al., 2004; Teramitsu et al., 2004; Mendoza et al., 2015). FoxP1-expressing cell types in rodents and birds include projection neurons and medium spiny neurons (MSNs) but neither neuroglia nor interneurons (Hisaoka et al., 2010; Precious et al., 2016; Garcia-Oscos et al., 2021). However, although it cannot be ruled out that some interneurons in both HVC and CMM express FoxP1, it is most likely that the largest proportion of FoxP1-expressing cells within each of these areas is accounted for by projection neurons (Garcia-Oscos et al., 2021).

HVC receives input from auditory areas including the CMM (Bauer et al., 2008), and projects to Area X and to areas of the song motor pathway (Mooney, 2000; for review, see Prather et al., 2017). HVC and CMM show increased neural activity in male and female zebra finches during conspecific song playbacks, and this neural activity is highest in response to familiar over unfamiliar song (Terpstra et al., 2004, 2006; Nick and Konishi, 2005; Kojima and Doupe, 2007; Ross et al., 2017; Van Ruijssevelt et al., 2017). Lesions of HVC in female canaries (Brenowitz, 1991; Del Negro et al., 1998) or CMM in female zebra finches (MacDougall-Shackleton et al., 1998) also impair conspecific song preference. The involvement of CMM and HVC in song preferences of female songbirds led us to hypothesize that high *FoxP1* expression in these areas might be important for auditory learning and memory maintenance (Riebel et al., 2002; Gobes and Bolhuis, 2007).

To test whether *FoxP1* expression in HVC or CMM is required for females to recognize and prefer songs, FoxP1 was knocked down in these areas either before (in juveniles) or after (in adults) the sensitive period for song preference learning (Riebel, 2003b, 2009). Subsequently, auditory memories were assessed in operant preference tests (Riebel, 2000; Holveck and Riebel, 2007). If expression of *FoxP1* in HVC or CMM was required for song memory formation or recall, knockdown and control groups should differ in their motivation to hear song, the consistency of choice and their preference strength for tutor song. Any differences between age groups and knockdown target areas can inform about locally or temporally-transient functions of FoxP1 in the development and maintenance of learned auditory preferences.

## Materials and Methods

### Subjects and housing

Subjects were 96 female zebra finches from the breeding colony at the Freie Universität Berlin. In the colony, breeding pairs were housed in 180 × 42 × 33 cm steel-wire cages with solid floors, wood chip bedding and equipped with a nest box and nesting material. Mobile perches from different materials and water baths were provided as enrichment. Most subjects (*N* = 79) were raised by their biological parents and stayed with them until 90 d posthatching (dph). The remaining females (*N* = 17) were also raised by their biological parents but moved to foster parents at age 15 dph, i.e., before the sensitive phase for song memorization (Roper and Zann, 2006), where they remained until 90 dph. All birds were provided with *ad libitum* water, cuttle bone, and tropical seed mix (Teurlings, Dordrecht) supplemented once a week with hardboiled egg and sprouted seeds. Bird rooms had a 12/12 h light/dark regime with a simulated dawn and dusk phase of 30 min each. Relative humidity was kept between 40% and 60%, and the ambient temperature was kept at 22°C. Birds received surgery at different ages (details below). As adults, birds were housed in groups of two to six individuals in cages of 120 × 90 × 90 cm until testing started. Enrichment was provided in the form of swinging perches, a mirror and a water bath, and bedding consisted of a sand grit mixture. Birds were kept under a 13/11 h light/dark schedule with a simulated dawn and dusk phase of 15 min each. Temperature was kept between 19°C and 22°C, with a relative humidity of 45–52%. Birds had constant access to water, cuttle bone, and tropical seed mix (Beduco, Schoten) supplemented once a week with hardboiled egg and freshly grated apple or carrot.

### Treatment groups

The four treatment groups were defined by when (as juveniles: 23 ± 2 dph or adults: 210 ± 124 dph) and where (HVC or CMM) they received the FoxP1 knockdown and labeled accordingly: HVC juvenile, HVC adult, CMM juvenile, CMM adult (for details, see below, Virus generation and Stereotaxic surgery). Because of a logistic cap on how many birds could be bred and treated simultaneously, not all experiments could be run in parallel. To prevent the timing of breeding from influencing the outcomes of the comparisons between treated females and matched controls, fledging females were assigned to a treatment and a matched control group on an alternating basis (assigning sisters to matched treatment and control groups wherever possible) until a sample size of *N* = 2 × 12 was reached for a treatment and its matching control group. Newly fledged females were then assigned to the next treatment and matched control group in the order of HVC adult, HVC juvenile or CMM adult and last CMM juvenile.

### Virus generation

Viral particles for injection were produced in Berlin as previously described (Haesler et al., 2007; Norton et al., 2019). Briefly, the virus was a pFUGW linker construct leading to the expression of a short-hairpin sequence and GFP, driven by the U6 promoter and the human ubiquitin C promoter, respectively (Lois et al., 2002). In total, three different constructs were prepared. Two of these constructs lead to expression of short-hairpins decreasing the expression of *FoxP1* via RNA interference and GFP as a marker of transduction (Norton et al., 2019). Two different shRNAs were used to reduce the impact of off-target effects on the behavioral analyses (Rossi et al., 2015; Song et al., 2015). The third construct was a nontargeting, GFP-expressing control. All sequences of the constructs are shown in Table 1.

**Table 1:** shRNA sequences of knockdown constructs used to downregulate FoxP1 expression as well as the control construct

	shRNA sequences, see also Norton et al. (2019)
Construct 1 (“*shY31*”)	5′-CCCCTATGCAAGCAATGCACCCAGTGCATG TCAAAGAAGAACCATTAGACCCAGATGAAA-3′
Construct 2 (“*shKRAK*”)	5′-CCAGATGAAAATGAAGGCCCACTATCCTTAGTGACAACAGCCAACCACAG-3′
Construct 3 (“*control*”)	5′-AATTCTCCGAACGTGTCACGT-3′

For a breakdown of different viral constructs used across groups, refer to Extended Data Table 1-1.

The experiments were run with one of 7 virus batches for each knockdown construct and one of five virus batches for the control construct, respectively. Each virus batch was used on an average of four birds (range 2–6) yielding an average of six (range 3–9) different virus batches per age/area treatment group plus matched control. This allowed maximal spread of virus batches across treatments and to obtain similar numbers of experimental and control animals within the same batch (for details see Extended Data Table 1-1). In every cohort, similar numbers of experimental and control subjects (ranging from 1 to 12 per virus batch) were reared together and received treatment or control injections with viral constructs of one batch within 7–14 d. Additionally, the two different knockdown shRNAs were divided equally among the cohorts to reduce the impact of off-target effects on the subsequent behavioral analyses (Song et al., 2015).

### Stereotaxic surgery

Before surgery, birds were caught individually from their home cage and weighed, and then received Rimadyl as analgesic (Pfizer, 5 ml/g bodyweight) intrapectorally, after which they were immediately returned to their home cage for 30 min until the analgesic took effect. The animals were then transferred in a mobile bird cage to the injection lab where they were anaesthetized with isoflurane (Dräger) via a beak mask. The initial level of isoflurane was between 3 and 4% (depending on the bird’s weight) and was subsequently lowered to 1.5–2% at a flowrate of 1l of O_2_ per minute.

As soon as a bird was deeply anaesthetized, it was fixed in a stereotactical apparatus (myNeurolab) connected to an injector (M-152, Narishige). All feathers at the back of the skull were removed with blunt tweezers and the area was sterilized with 70% EtOH. Subsequently, an ∼4-mm horizontal incision was placed into the skin to allow for a longer vertical cut of the skin of ∼1 cm. Within this opening, a rectangular piece (∼1.5 × 1.5 mm) of the skull bone was dissected and pushed under the surrounding skin to prevent it from drying out. The opening in the skull was located around the bifurcation of the midsagittal sinus which was optically determined after bone removal. The dura mater was kept in place and only punctured locally with the injection glass capillary (30-μm tip), which was used to inject 0.25 μl of virus with a titer of >1 × 6^10^ particles per microliter bilaterally into each injection site (Table 2) based on coordinates determined by injections using FluoSpheres (F8842, Thermo Fisher Scientific) diluted 1:10 in 1× phosphate buffered saline. Previous experiments using this method showed that although fluorescent beads and viral particles may spread differently, the overall targeting was comparable (Haesler et al., 2007; Norton et al., 2019). As female HVC is relatively small, compared with the same region in males, viral constructs were injected as superficially as possible to avoid injections into the underlying shelf. Still, and despite all precautionary measures, HVC shelf might have been targeted as well.

**Table 2:** Coordinates for viral injections (mm)

Direction/group	Juvenile HVC	Adult HVC	Juvenile CMM	Adult CMM
Anterior/posterior	0 / 0.15	0/0.2	1.4/1.5	1.5/1.6
Medial/lateral	1.9	2	1.1/1.0/0.9/0.8	1.2/1.1/1.0/0.9
Dorsal/ventral	0.35/0.25	0.4/0.25	0.65	0.7

Medial/lateral coordinates are indicated as negative and positive for the left and right hemisphere, respectively.

After each injection, the glass capillary was kept in place for 30 s to allow pressure to normalize around the injection site before moving to the next site. For each operating session, the first injected hemisphere was chosen pseudo-randomly and subsequently left and right hemispheres were injected alternatingly. After the injections, the bone was moved back into place and the skin incision was closed by overlapping its edges and gluing it with Collodion (nitrocellulose). As soon as the incision site was fully closed, isoflurane was reduced to 0% and the oxygen level increased to 2% to cancel anesthesia until the bird was fully awake (range = 39–103 min, average = 57 min after anesthesia was initiated). Birds were then returned to the colony and checked every hour during the rest of the day. Adult subjects were returned to all-female aviaries (2 × 2 × 3 m, with *N* = 15–30 birds per aviary). Juveniles were returned to their (foster-) parents and siblings and remained in their family group until 90 dph to be then moved to all-female aviaries. All birds were seen to move, eat, fly and socialize within 1 h after surgery and were behaviorally indistinguishable from nonoperated birds the day after surgery.

### Stimulus songs

**Figure 1.**
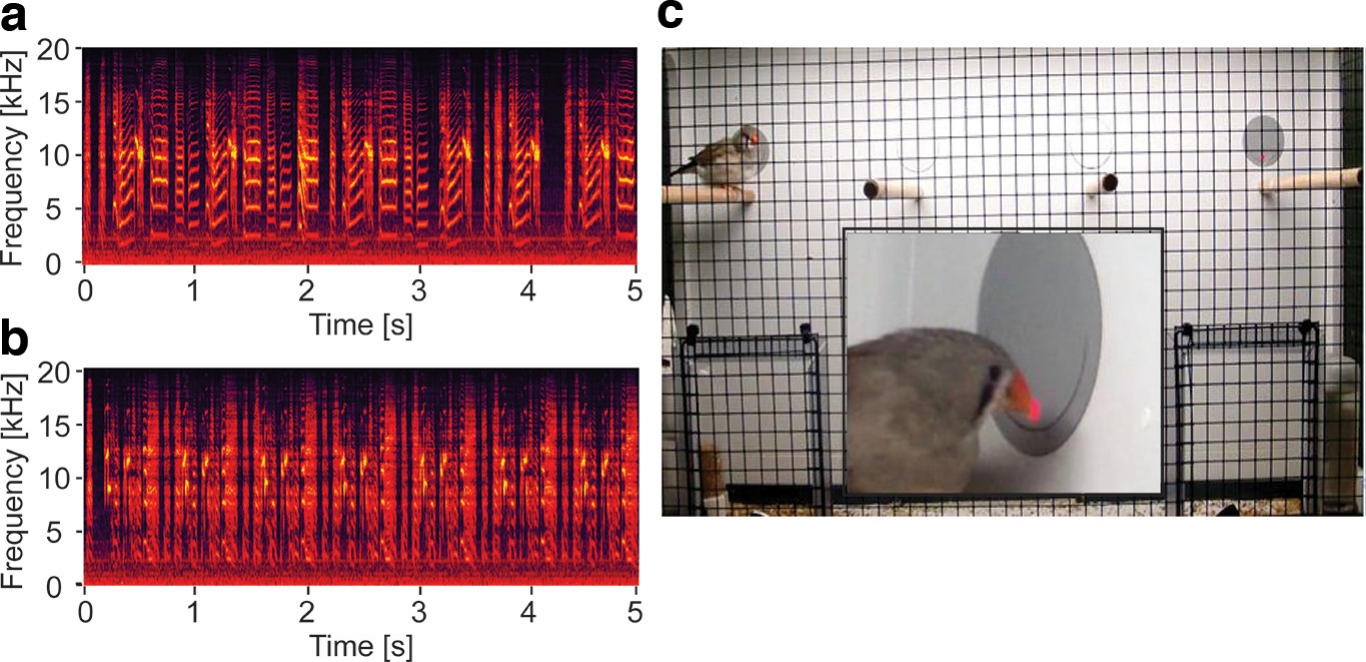
An example of a stimulus set used for playback and the testing setup. ***a***, ***b***, Two spectrograms (frequency over time) of two song stimuli used in the preference test. Color levels indicate local power distribution, the brighter the color the higher the amplitude at a specific location. ***c***, Shows a female zebra finch in the operant preference test setup. One of the gray pecking disks with the corresponding red LED light is visible in the top right corner, the second pecking key is partly obscured by the female on the outer left perch. The central inset shows a close up of a female pecking one of the keys. Refer to Extended Data Figure 1-1 for detailed properties of the different stimuli used during the preference tests.

Following established protocols for song preference testing (Riebel, 2000; Riebel et al., 2002; Holveck and Riebel, 2010, 2014), stimulus sets consisting of the song of the female’s father and an unfamiliar male were assembled as follows: songs of all (foster-)fathers were recorded when the pair was not breeding. For recording, males were first transferred individually from their home cage (90 × 35 × 45 cm) to a recording cage (40 × 30 × 40 cm) in a sound attenuated chamber (60 × 60 × 80 cm) in the afternoon to acclimatize. Recordings started the following morning until several long bouts of song were obtained. If a bird did not sing during the first morning it was kept in the recording chamber for an additional morning. Song was recorded with cardioid microphones (ME 64, Sennheiser) mounted in front of the cage at a 20-cm distance from the perches and written directly onto a hard disk (Aardvark Direct Pro Q10 soundcard, sampling rate 44.1 kHz, 16 bits) using SAP software version 2011 (Tchernichovski et al., 2000) with automatic energy detection settings for 2–10 kHz, detection limits between 3 and 60 s, and a buffer of 5 s. Recordings were screened using spectrograms (sample rate 44.1 kHz, FFT size 1024 bits, step size 0.1 μs, frequency resolution 0.0001 Hz, time resolution 0.1 ms, 20-kHz bandwidth, Blackman window, produced with the software Syrinx 2.6 h, John Burt, University of Washington, Seattle, WA) to visually identify the most frequent motif of each male, defined as the most common sequence of syllables in ten song bouts. For each male, a song with four to seven repetitions was selected. The songs of the females’ respective (foster) fathers served as “familiar” stimuli, while the songs of other fathers were used as “unfamiliar” stimuli. Familiar-unfamiliar stimulus sets were formed by matching pairs of songs that were as similar as possible in the number of syllables (average ± SD 4.8 ± 0.6) and motif repetitions (5.4 ± 0.9), as well as in overall song duration (Fig. 1*a*,*b*). The selected songs ranged from 5.0 to 6.87 s in duration (average: 5.72 ± 0.51 s) but within matched pairs total duration did not differ by >5.2%. Where possible (*N* = 63/96 birds), each stimulus set was used for the daughters of both males that contributed the songs. Playbacks for the remaining 33 birds (juvenile HVC: one control, three knockdowns; adult HVC: six controls, five knockdowns; juvenile CMM: four controls, two knockdowns; adult CMM: five controls, seven knockdowns) consisted of the respective females’ fathers’ songs and the unfamiliar song which matched best in duration and number of elements and motifs. This design ensured that each song was offered equally often (and in the same combination) as familiar and as unfamiliar song (for complete list and details, see Extended Data Fig. 1-1).

10.1523/ENEURO.0328-22.2023.f1-1 Extended Data Figure 1-1 Test stimuli during preference tests of all birds. Bird IDs with an asterisk indicate playbacks that were used for multiple stimulus pairings. Download Figure 1-1, DOC file.

10.1523/ENEURO.0328-22.2023.tab1-1 Extended Data Table 1-1 Viral constructs which were used during this study resulting in different virus batches distributed among the experimental groups. Indicated are the experimental condition they belong to, either control or knockdown, the serotype of one control construct and two knockdown versions of a short-hairpin construct, their respective production date and area, age group, and number of birds they were injected into. Download Table 1-1, DOC file.

### Operant song preference tests

Behavioral testing started 14–28 d after the birds had been moved to their group cages. Birds from the juvenile groups were between 108 and 137 d old (mean ± SD 119 ± 8) and those from the adult groups 116–538 d old (mean ± SD 246 ± 137). For preference testing a validated operant song preference paradigm was used (Riebel, 2000; Holveck and Riebel, 2007), taking advantage of the positively reinforcing qualities of song. Briefly, females learned that pecking the operant keys elicited song playbacks. Key-pecking was voluntarily, and throughout testing, birds had *ad libitum* access to water, food, and grit. To start training, individual females were moved into an experimental cage (the “Skinner box”; see Fig. 1*c*) in one of 20 sound attenuated chambers (minimum size 2.4 × 1.4 × 2.3 m) between 3 and 5 P.M. The experimental cages (70 × 30 × 45 cm) were made from wire mesh but for the floor and a solid plywood back panel with two integrated pecking keys (a 5-cm diameter piezoelectric plate with an embedded 5-mm diameter red LED at the bottom). The pecking keys were connected to custom made control devices (Leiden University electronics workshop) containing Oki MSM6388 soundchips (Tokyo) which were individually controllable via laptops (Sony Vaio E series, Sony) from outside the training chambers via custom software (Leiden University electronics workshop). The laptops that were connected to the Skinner boxes controlled the playbacks from a loudspeaker (Vifa 10BGS119/8) suspended from the ceiling at 1 m above the center of the cage. The custom written software kept a data log of occurrence and time of each key peck and the associated playbacks. Sound amplitude of stimuli was adjusted to peak levels of 70 dB re 20 μPa (set to continuous fast measurements over 5 s, RION NL 15) at the perches near the pecking keys.

To start training, females were first left to acclimatize to and explore the new environment for 1–2 d (41± 3 h), as earlier work with this setup showed that ∼30% of females discover that key pecking triggers song playback by autoshaping (Holveck and Riebel, 2014). Therefore, during this phase, the red LEDs on the pecking keys were switched on continuously and the setup was operational during hours with lights on to provide immediate feedback should a bird start pecking the keys. To avoid exposing the females to the test stimuli before testing started, pecking either key during this phase triggered playback of the song of an unfamiliar male zebra finch until the females were actively pecking each key at least 10 times per day. A total of 46/96 birds reached this criterion during the initial combined acclimatization and autoshaping phase of 1–2 d (juvenile HVC: 7 of 12 birds in the control group, 6/12 knockdown; adult HVC: 4/12 control, 6/12 knockdown; juvenile CMM: 7/12 control, 7/12 knockdown; adult CMM: 4/12 control, 5/12 knockdown). The birds that had not reached criterion at this stage (50/96) received two 20-min training sessions per day between 9 and 11 A.M. and 3 and 5 P.M. for a maximum of five training days. Training used stepwise shaping by the experimenter by rewarding the bird with song for approaching a key, moving their head toward the pecking key or touching the area around the key with their beak (Holveck and Riebel, 2014). Birds that had not reached criterion after 5 d (19/96) were returned to their home cages, and after a 7-d resting period moved back into the Skinner box setup to start the training cycle again. The birds that still had not reached criterion after two training sessions (9/96), received a second resting period and a third training cycle, now with seeds of Japanese millet (*Echinochloa esculenta*) fixed with clear tape on top of the pecking keys. All remaining birds reached criterion this way.

The actual preference tests started the day after the birds reached criterion. The Skinner box was now programmed such that pecking of one key resulted in playback of the song of a female’s tutor (the father or foster-father which was present between 23 and 90 dph) or an unfamiliar song (the tutor song of another experimental female which was tested with the same stimulus combination in a matched-pairs design). This way each song was tested as a familiar song for one female and as an unfamiliar stimulus for another female. Assignment of stimulus songs was pseudo-random on the first day of testing and afterward songs were swapped between the pecking keys every 24 h (during lights off). This way, each stimulus was presented an equal number of times at either side of the cage during the 4-d-long preference tests, thus controlling for potential individual side preferences that could confound song preferences.

### Brain extraction

After the experiments were completed, females were returned to their home cages where they were housed with two to five familiar females for at least one week. Between 3 and 5 P.M. on the day before brain extraction, birds were individually transferred into familiar sound attenuated chambers. The next morning (between 6:30 and 6:50 A.M.), the birds were killed with an overdose of isoflurane gas and subsequently their brains were extracted before lights went on to minimize activity dependent expression changes. Age at brain extraction for juvenile groups was between 179 and 210 dph and between 165 and 579 dph for adult groups. Hemispheres were separated along the midline and frozen in Tissue Tek Optimal Cutting Temperature Compound (OCT, Sakura) on dry ice and stored at −80°C until further processing.

### RNA extraction

To determine the extent to which the injection of shRNA reduced the FoxP1 expression, quantitative PCR (qPCR) of the targeted tissue was performed. To do so, RNA was extracted from brain punches. For this, brain hemispheres were first embedded in OCT and sliced sagittally in a cryostat into 200-μm sections. For each section, HVC or CMM was manually punched out with biopsy punchers (0.35–0.75 mm in diameter, WPI) and immediately submerged in RNAlater (AM7021, Thermo Fisher Scientific) to prevent RNA degradation. The remaining slice was fixed in 4% fresh, ice-cold paraformaldehyde (PFA) to validate the punching site (see below, Validation of correct targeting).

Correctly punched tissue, determined by GFP fluorescence examined under a stereo microscope and immunohistochemical profile (see below) was pooled by hemisphere and RNA was extracted with a column-based RNA extraction kit for low amounts of tissue following the protocol (RNeasy micro plus, QIAGEN). RNA concentration was quantified with a Bioanalyser RNA kit (Bio-Rad) system and extracted RNA was stored on −80°C until further use. After RNA extraction and quantification, 10 ng of each sample were used for reverse transcription. Superscript III enzyme kit (Thermo Fisher Scientific) was used according to the manufacturer’s protocol. cDNA was kept frozen on −20°C until further use. Thawed cDNA samples were diluted 1:5 in molecular grade water before qPCR. A total of 2 μl of cDNA sample were mixed with 2 μl of molecular grade water, 5 μl of iQ SYBR Green Supermix (Bio-Rad) and 0.5 μl of 600 nm forward and reverse primers, respectively (for a detailed list of used primers, see Extended Data Fig. 3-1). The PCR cycling conditions were as follows: 300 s at 95°C, 40 cycles of 30 s 95°C, 30 s 60°C, 45 s 95°C. Hydroxymethylbilane synthase (HMBS) served as a housekeeping gene to normalize gene expression (Olias et al., 2014). All samples were run in triplicate to generate average Cq values.

10.1523/ENEURO.0328-22.2023.f3-1 Extended Data Figure 3-1 Primers used for qPCR validation of knockdown in RNA extracted from zebra finch target regions HVC and CMM. Download Figure 3-1, DOC file.

### Validation of correct targeting

**Figure 2.**
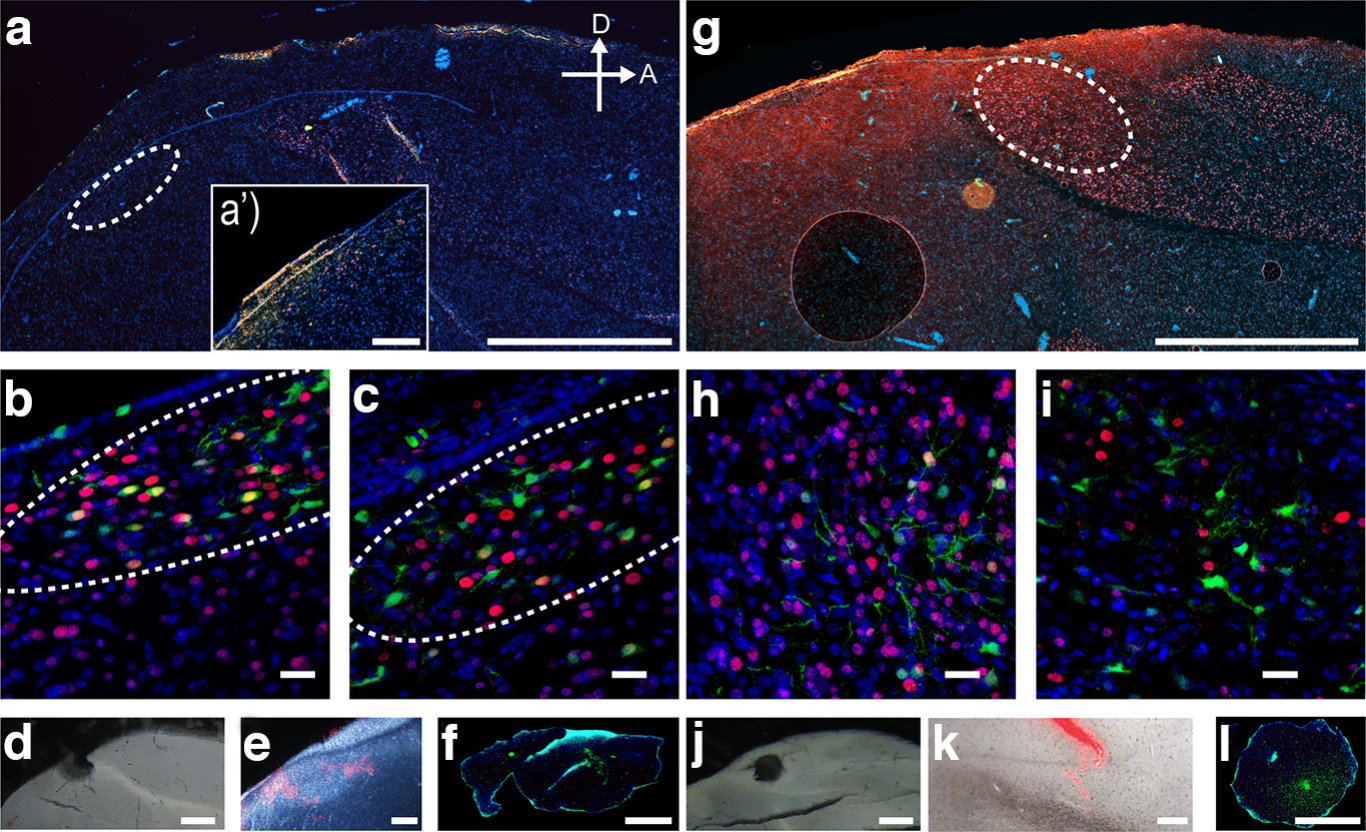
Immunohistochemistry of an HVC (***a–c***) and CMM of adult-injected birds (***g–i***) after the experiments were completed. Shown are merged stainings of GFP (green) indicating virus-infected neurons, FoxP1 immunoreactivity (red) and a nuclear Hoechst stain (blue). ***a***, Stitched sagittal overview indicating FoxP1 expression throughout the brain of an HVC-injected bird, HVC outlined by dashed line. Inset ***a’*** shows a magnified version of FoxP1-positive cells in female HVC, highlighted by zRalDH stainings in orange. ***b***, Close-up of the injected area within HVC of a bird injected with the control construct, dashed line indicates HVC. ***c***, Close-up of the injected area in HVC of a bird injected with a knockdown construct, dashed line outlines HVC. Note, that a successful expression of the knockdown construct does not fully eliminate FoxP1 immunostaining and that the intensity of GFP expression varies across cells and stainings, thus micrographs are only for visualization not for quantification as the majority of tissue was collected for RNA extraction, additional projects and validation of the knockdown via qPCR. ***d***, Validated punching site of HVC after experiments were completed. ***e***, Dark-field image of previously injected fluorescent beads into HVC to validate coordinates used for viral injections. Dashed outline indicates HVC. ***f***, Confirmation of GFP-fluorescence within the extracted tissue punch of HVC. ***g***, Stitched sagittal overview indicating FoxP1 expression throughout the brain of a CMM-injected bird, dotted line indicates CMM. ***h***, Close-up of the injected area within CMM of a bird injected with the control construct. ***i***, Close-up of the injected area within CMM of a bird injected with a knockdown construct. ***j***, Validated punching site of CMM after experiments were completed. ***k***, Bright-field image of previously injected fluorescent beads into CMM to validate coordinates used for viral injections. Dotted outline indicates position of CMM. ***l***, Confirmation of GFP-fluorescence within the extracted tissue punch of CMM (for additional close-ups and overviews of the juvenile groups see Extended Data Fig. 2-1*f–i’*). Scale bars: 1000 μm (***a***, ***d***, ***g***, ***j***), 20 μm (***b***, ***c***, ***h***, ***i***), and 200 μm (***a’***, ***e***, ***f***, ***k***, ***l***). Tile scans of the brain slices corresponding to ***b***, ***c***, ***h***, and ***i*** are shown in Extended Data Figure 2-1*b–e*, respectively.

The tissue surrounding the punch was examined for the correct location. Additionally, after each 200-μm slice used for punching, an 8-μm slice was cut for immunohistochemical detection of GFP expression in HVC or CMM. Thin 8 μm slices were thawed and fixed for 10 min in fresh, ice cold 4% PFA on 4°C, and kept in the dark at 4°C until further processing. After fixation, immunohistochemical staining for GFP, Hoechst, and FoxP1 was conducted to validate the injection site of the virus. Some slides were additionally incubated with a zRalDH antibody to confirm the outlines of HVC (Fig. 2*a'*; Roeske et al., 2014). Note that stainings and imaging could only be conducted on a subset of slices as the majority of tissue was collected for qPCR and RNA analyses.

10.1523/ENEURO.0328-22.2023.f2-1 Extended Data Figure 2-1 Immunohistochemistry of HVC or CMM-injected birds after completion of the experiments. Shown are merged stainings of GFP (green) indicating virus-infected neurons, and FoxP1 immunoreactivity (red). ***a–a”***, Sagittal images from three different females highlighting the difference of *FoxP1*-expressing cells in HVC (dashed line). Filled arrowheads in ***a*** and ***a”*** point towards FoxP1-positive cells ventral from HVC that might be located in the HVC-shelf. Empty arrowheads in ***a’*** show *FoxP1*-expressing neurons in HVC. ***b–e***, Sagittal scans of the corresponding high magnification images (dashed squares) of females injected as adults shown in Figure 2*b*,*c*,*h*,*i*, respectively. ***b***, ***d***, Slices from females injected with control constructs. ***c***, ***e***, Slices from females injected with knockdown constructs, respectively. ***f***, Close up of HVC from a bird injected into HVC with the control construct as juvenile. ***f’***, Sagittal overview of the brain slice corresponding to ***f***. Dashed outline highlights the coordinates of ***f***. Arrowheads indicate cells expressing both *GFP* and *FoxP1*. ***g***, Close up of HVC from a bird injected into HVC with a knockdown construct as juvenile. ***g’***, Sagittal overview of the brain slice corresponding to ***g***. Dashed outline highlights the coordinates of ***g***. Arrowheads indicate cells expressing *GFP* but not *FoxP1*. ***h***, Close up of CMM from a bird injected into CMM with the control construct as juvenile. ***h’***, Sagittal overview of the brain slice corresponding to ***h***. Dashed outline highlights the coordinates of ***h***. Arrowheads indicate cells expressing both *GFP* and *FoxP1*. ***i***, Close up of CMM from a bird injected into CMM with a knockdown construct as juvenile. ***i’***, Sagittal overview of the brain slice corresponding to ***i***. Dashed outline highlights the coordinates of ***i***. Arrowheads indicate cells expressing *GFP* but not *FoxP1*. Scale bars: 200 μm (***a–a”***), 1000 μm (***b–e***, ***f’***, ***g’***, ***h’***, ***i’***), and 20 μm (***f***, ***g***, ***h***, ***i***). Download Figure 2-1, TIF file.

### Immunohistochemistry

To validate the localization of the injected virus construct, triple immunohistochemistry analyses were conducted on cryostat sections. Slices were fixed in 4% PFA in 1× PBS for 10 min at 4°C and blocked in 10% ROTI Histol (Carl Roth) solution between stainings. The following antibodies were used: a mouse monoclonal (JC12) antibody against FoxP1 (1:100, ab16645, Abcam, Cambridge), a goat antibody against zRalDH (1:50, sc.22 591, Santa Cruz, Dallas) to delineate HVC, and a rabbit GFP antibody (1:100, ab6556, Abcam, Cambridge) to increase signal strength from virally transmitted GFP. Ultimately, slices were counterstained with Hoechst (Sanofi) and mounted for fluorescence imaging (Apotome, Zeiss).

### Statistical analyses

The total amount of keypecks (activity) and the proportion of keypecks for the tutor song (preference strength) were used as primary response variables to gauge motivation to hear song and as a validated measure of song memorization (Riebel, 2000; Terpstra et al., 2006; Holveck and Riebel, 2007). Consistency of females’ behavior was assessed by calculating the repeatability of these two variables over the first and second block (day 1–2 and day 3–4, respectively) of the 4-d preference tests. As these measures were highly repeatable across the two blocks (for details, see Results), data from days 1 to 2 and days 3 to 4 were subsequently pooled to calculate one overall preference and one activity value based on the total keypecks during the 4-d tests for all subsequent analyses.

Preference strength for familiar versus unfamiliar song was calculated by dividing the total number of pecks for the familiar song by the total number of keypecks as follows: $\frac{\sum_{i-k}keypecks\;familiar\;song}{\sum_{i-k}keypecks\;total}$ (i = first, k = last day of the preference test). To ensure properties of normal distributions for analyses, all preference values were arcsine of the square root transformed (recommended transformation for proportional data that are bound between 0 and 1 centered around the mean) while keypecks were base 10 logarithmically transformed to adjust for the right skew of the data.

Subsequently, data were checked and confirmed to be normally distributed using Shapiro-Wilk tests (preference: W(96) = 0.97, *p* = 0.06; keypecks: W(96) = 0.98, *p* = 0.08; Shapiro and Wilk, 1965) in R v3.5 (R Development Core Team, 2011).

Keypecking was compared between knockdowns and controls per treated brain area (CMM, HVC) using generalized linear mixed models (glm2, v1.2.1; Marschner, 2011) based on Gaussian distributed data. Preference strength for tutor song in all treatment groups was likewise analyzed separately for HVC-injected and CMM-injected groups, using GLM assuming a binomial distribution of data. To test whether preference strength deviated from a 0.5 chance level, 0.5 was subtracted from each female’s preference value (if females have no preference, proportions of pecks will be 0.5 for both the familiar and unfamiliar song). The values for the deviation from a 0.5 chance level were then used as response variable of a mixed linear model (lme4; Bates et al., 2015) with only random effects (bird ID, virus batch) to test whether the intercept deviated significantly from 0 (model A) which corresponds to a significant preference for one song category. Subsequently, it was tested whether females with different treatment (knockdown or control) differed in preference strength by adding treatment as a fixed factor (model B). The next hypothesis assumed that age at FoxP1 knockdown has an effect by adding age at treatment (juvenile or adult) as an additional fixed factor and also investigated the impact of an interaction between age and treatment to account for behavioral variability (model C). Models to determine whether the efficiency of the FoxP1 knockdown was predictive of pecking activity or preference strength were also based on GLM with Poisson distributed data. These models included virus batch as random effect, relative FoxP1 expression in comparison to the respective matching control group, age and area of injection as well as their interaction as fixed factors. As the dependent variable, total number of pecks or preference strength for familiar song were included.

For *post hoc* testing of the models, two sample *t* tests of controls and knockdowns of specific groups were conducted where necessary. Multiple *t* tests were corrected for false discovery rate (Benjamini and Hochberg, 1995). ANOVAs were conducted to determine the knockdown efficiency across treatment groups, as well as possible effects of knockdowns on learning speed. *Post hoc* correction was conducted with Tukey’s multiple comparison test (Tukey, 1949).

### Ethical statement

All experimental procedures were approved by the veterinary department of the Freie Universität Berlin and by the ethics committee of the Regional Office for Health and Social Affairs Berlin (LAGeSo) under REG 0019/15. All experiments at Leiden University were approved by the Animal Experimentation Committee at Leiden University (DEC license 14234) and by a license of the Ministry of Infrastructure and Environment (GGO license 14-097) in accordance with Dutch laws.

## Results

### Validation of knockdown and localization of the virus construct

Immunohistochemistry analyses showed localized expression of GFP that was restricted to the target areas of HVC (Fig. 2*a–c*; Extended Data Fig. 2-1*a–a”*,*b*,*c*,*f*,*g*) and CMM (Fig. 2*g–i*; Extended Data Fig. 2-1*d*,*e*,*h*,*i*). However, FoxP1 expression indices ([FoxP1 signal/background signal] × ([FoxP1+ nuclei/Hoechst+ nuclei]) did not differ between knockdowns and controls of any group (Wilcoxon rank sum *p* > 0.05). We next quantified the normalized fraction of cells expressing GFP and FoxP1 in a subset of HVC-injected birds (control and knockdown *N* = 5 each). In relation to controls, the fraction of GFP and FoxP1 coexpressing cells in knockdowns varied from 34.9% to 88.6% in knockdown birds. On average, the fraction of double-labeled cells in knockdowns was 56.7 ± 8.2% (*p* = 0.008, Wilcoxon rank sum) in relation to double-labeled cells in controls. Tissue punches (Fig. 2*d*,*j*) from targeted areas that were previously validated (Fig. 2*e*,*k*) were visually confirmed to show fluorescence (Fig. 2*f*,*l*).

**Figure 3.**
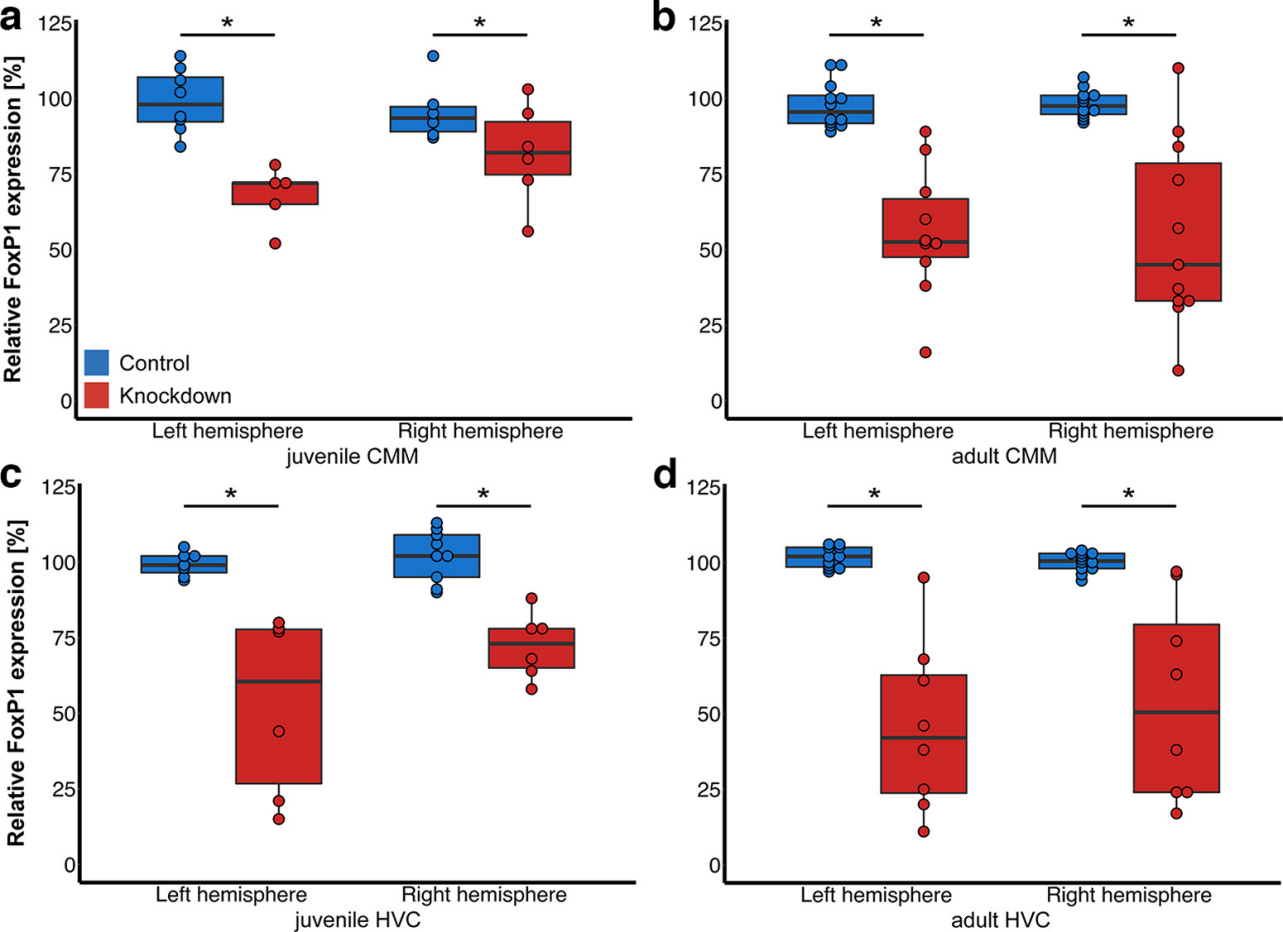
qPCR-based analysis of knockdown efficiency. Boxplots depict relative expression levels of FoxP1 in relation to normalized expression of HMBS in the knockdown and control groups separately for each hemisphere. ***a***, Juvenile CMM. ***b***, Adult CMM. ***c***, Juvenile HVC. ***d***, Adult HVC. Boxes indicate the interquartile range from first to third quartile, the line indicates the median, whiskers show the 1.5-fold interquartile range. Overlaying points depict data from individual birds. Significant differences of *p* < 0.05 are indicated by an asterisk. Primers used for qPCR analyses are shown in Extended Data Figure 3-1.

The knockdown efficiency according to qPCR of FoxP1 of GFP-positive tissue varied from 10% to 70% across individual birds. Quantification of gene expression via qPCR showed that *FoxP1* expression in HVC and CMM was significantly lower in all treatment groups compared with controls across both hemispheres (*F*_(1,96)_ = 176.57, *p* < 0.001, two-way ANOVA with area and developmental stage as factors; Fig. 3) independent of the injected hemisphere (*F*_(1,96)_ = 1.64, *p* = 0.2) or area (*F*_(1,96)_ = 0.37, *p* = 0.54). There was however a significant age*treatment interaction: in birds injected into CMM as juveniles the knockdown showed reduced efficiency (*F*_(1,96)_ = 5.07, *p* = 0.03). Despite significant differences on a group basis, some knockdown birds showed higher *FoxP1* expression than matched controls.

### Learning speed

There was no difference in how many days with or without training the birds needed to successfully peck the keys for song reward among any of the treatment groups (knockdowns: mean ± SD 3.7 ± 3.4 d to criterion, controls: 4.2 ± 3.9 d to criterion, two-way ANOVA *F*_(1,95)_ = 0.01 *p* = 0.98). Neither treated area (*F*_(1,95)_ = 0.65 *p* = 0.42) nor the birds’ age during the injection (*F*_(1,95)_ = 2.1 *p* = 0.16), nor an interaction between age and injected area (*F*_(1,95)_ = 0.001 *p* = 0.97) affected the number of days to reach criterion.

Similar to the number of days, the required training sessions in case a bird did not start to peck the provided keys on its own did not differ between knockdowns and matched controls (knockdowns: 3.4 ± 5.6 sessions, controls: 4.8 ± 7.4 sessions, two-way ANOVA *F*_(1,95)_ = 0.03 *p* = 0.86). Neither were necessary training sessions affected by the treated area (*F*_(1,95)_ = 1.52 *p* = 0.22) nor by the birds’ ages at the time of injection (*F*_(1,95)_ = 1.37 *p* = 0.25) or the interaction between age and injected area (*F*_(1,95)_ = 0.029 *p* = 0.87).

### Repeatability

**Figure 4.**
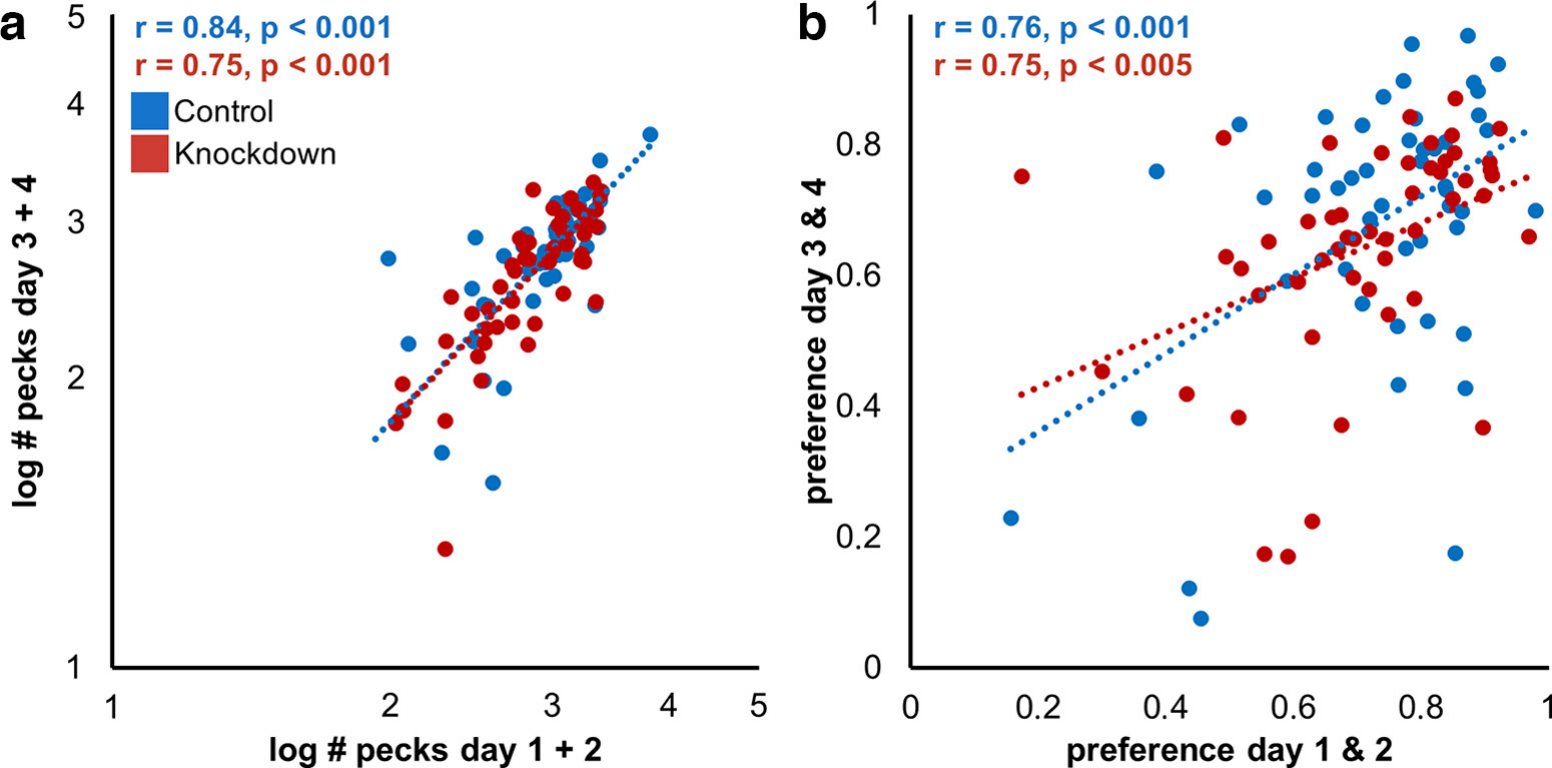
Individual behavior of birds was highly repeatable across preference testing days. Scatterplots for pecking (***a***) and preference (***b***) behavior of all tested animals across days. Linear regressions across all controls or knockdowns are indicated by dotted lines.

The comparison of the first versus the second block (i.e., day 1–2 vs day 3–4) of the actual preference tests showed females in both control and experimental groups to be consistent in their pecking activity and preferences. Total number of keypecks (Fig. 4*a*) and preference strength (Fig. 4*b*) for familiar song were highly repeatable between block 1 and block 2 (keypecks controls: Pearson’s *r*(48) = 0.84, *p* < 0.001, knockdowns: *r*(48) = 0.75, *p* < 0.001; preference controls: *r*(48) = 0.76, *p* < 0.001, knockdowns: *r*(48) = 0.75, *p* < 0.005). Further analyses were thus conducted with the totals of days 1–4.

### Pecking activity

**Figure 5.**
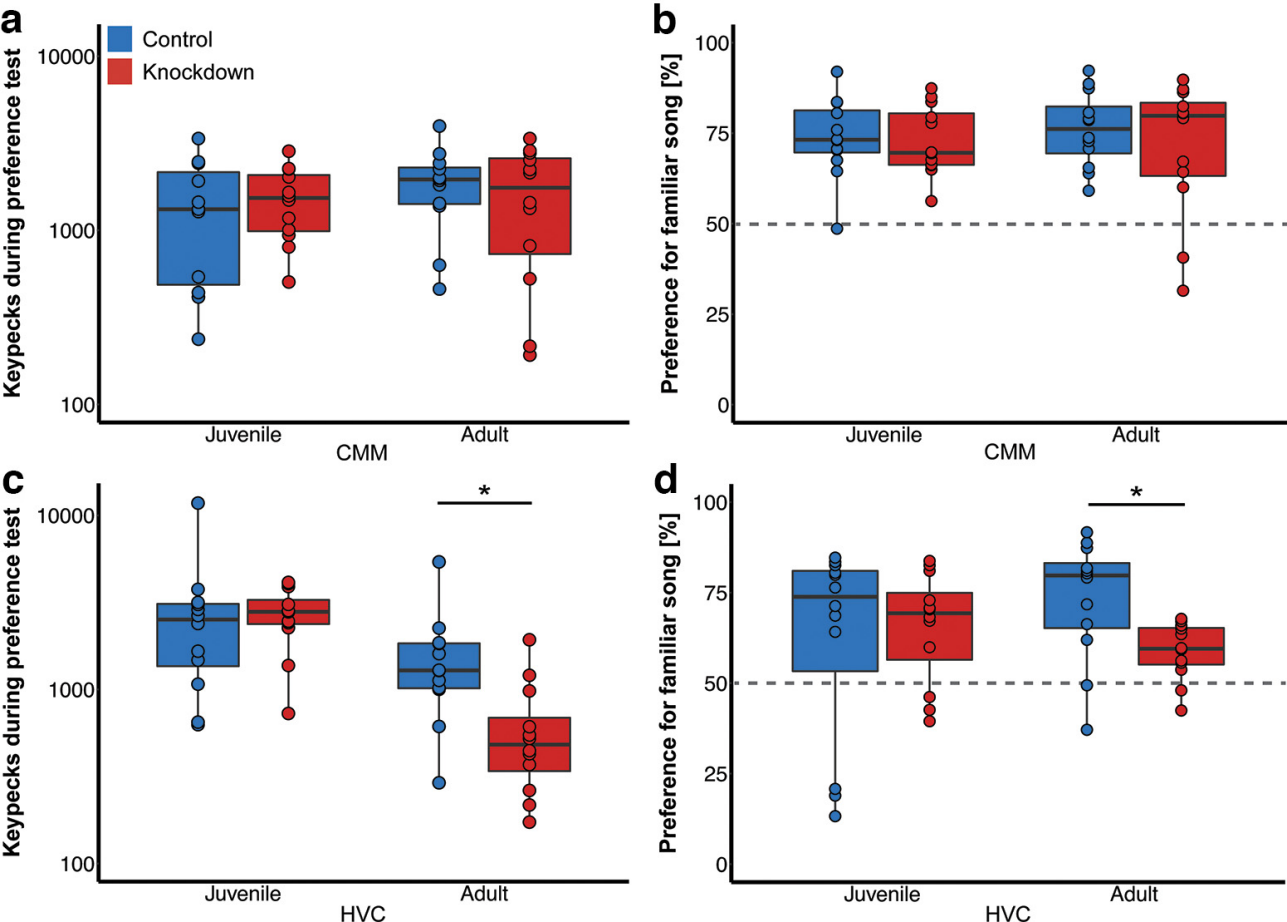
Behavioral data of all birds during preference tests. Boxplots (same convention as in Fig. 3) show individual keypecks (***a***, ***c***) and preference for familiar song (***b***, ***d***). Overlaying points depict data from individual birds. Significant differences of *p* < 0.05 are indicated by an asterisk. For detailed statistics on pecking behavior refer to Tables 3 and 4. Statistics on preference behavior are shown in Tables 5 and 6. ***a***, Total number of keypecks (sum test-days 1–4) of birds injected into CMM with control or knockdown constructs. ***b***, Preference strength for familiar song per treatment group of birds injected into CMM. Dotted line indicates chance level. ***c***, Total number of keypecks (sum test-days 1–4) of birds injected into HVC with control or knockdown constructs. ***d***, Preference strength for familiar song per treatment group of birds injected into HVC.

During the 4 d of preference tests, females of all treatments (*N* = 96) initiated song playback by keypecking for a total of on average 1811 ± 1514 times over the 4-d testing period (range: 91–11,767, for group averages see Table 3). Birds that had received the viral FoxP1 knockdowns in CMM either as juveniles or adults did not differ from the controls in pecking activity (GLM total number of keypecks, see Table 4; Fig. 5*a*). The best fitting model for pecking behavior of CMM-injected birds contained neither treatment nor age and both factors contributed minimally to the model’s weight. Birds that had received the viral FoxP1 knockdown injections into HVC showed a significant interaction of treatment and age in the best model fit (see Table 5; Fig. 5*c*) suggesting that both factors significantly contributed to the pecking activity, as models without both factors had a lower weight. *Post hoc* analyses revealed that this effect resulted from adult birds with a FoxP1 knockdown in HVC pecking significantly fewer times than their matched controls (two-sample *t* test *t*_(24)_ = 2.67, *p* = 0.015; Fig. 5*c*). This difference was not observed in the females that had received knockdowns in HVC as juveniles (two-sample *t* test *t*_(24)_ = 0.22, *p* = 0.39).

**Table 3:** Preference for familiar song and keypecking activity across different treatment groups

Area	Age	Treatment	Preference % ± SD	*t*_(24)_	*p*	Keypecks ± SD	*t*_(24)_	*p*
HVC	Juvenile	Control	62.1 ± 7.6	0.37	0.367	2932 ± 820	0.22	0.385
		Knockdown	65.4 ± 4.2			2736 ± 282		
	Adult	Control	73.1 ± 4.6	**2.67**	**0.015**	1632 ± 361	**2.45**	**0.024**
		Knockdown	58.7 ± 2.2			641 ± 140		
CMM	Juvenile	Control	73.7 ± 3.1	0.2	0.387	1330 ± 289	0.6	0.328
		Knockdown	72.8 ± 2.7			1544 ± 193		
	Adult	Control	76.1 ± 2.9	0.9	0.261	1920 ± 258	0.4	0.363
		Knockdown	76.4 ± 5.3			1753 ± 32		

Pairwise comparisons for knockdowns and their matched controls are corrected for multiple testing.

**Table 4:** GLM results for pecking activity of the groups that either received a viral knockdown or sham treatment in CMM

CMM pecking	Estimate	Std. error	*t* value	*p* value
Model A^*1*^ Total pecks ∼ (1|Bird ID) + (1|Virus batch)				
Intercept	3.10	0.05	58.42	**<0.001**
Model B: Total pecks ∼ (1|Bird ID) + (1|Virus batch) + Treatment				
Intercept	3.09	0.09	36.50	**<0.001**
Treatment: knockdown	0.02	0.12	0.20	0.39
Model C: Total pecks ∼ (1|Bird ID) + (1|Virus batch) + Treatment + Age/Treatment × Age				
Intercept	3.14	0.10	33.88	**<0.001**
Treatment: knockdown	0.03	0.11	0.30	0.38
Age: adult	0.11	0.11	−1.06	0.23
Age × Treatment	0.07	0.23	−0.68	0.31
				
Models	AIC	ΔAIC	Weight	
Model A	49.2	0.0	0.89	
Model B	53.7	4.5	0.09	
Model C	57.2	8.0	0.02	

Shown are the null model (A), the Treatment model (B), and a model considering Treatment and Age (C). Indicated are estimate, SE (std. error), and the respective *t* and *p* values of the intercept and the included fixed factors. To find the best fitting model, the Akaike information criterion (AIC), and the weight of each model were calculated and models were ordered from best to worst fit.

1 bird ID and virus batch are included as random effects in all models. Significant *p* values are marked in bold.

**Table 5:** GLM results for pecking activity of the groups that either received a viral knockdown or sham treatment in HVC

HVC pecking	Estimate	Std. error	*t* value	*p* value
Model A^*1*^ Total pecks ∼ (1|Bird ID) + (1|Virus batch)				
Intercept	3.10	0.10	32.48	**<0.001**
Model B: Total pecks ∼ (1|Bird ID) + (1|Virus batch) + Treatment				
Intercept	3.23	0.15	20.96	**<0.001**
Treatment: knockdown	−0.21	0.20	−1.05	0.23
Model C: Total pecks ∼ (1|Bird ID) + (1|Virus batch) + Treatment + Age/Treatment × Age				
Intercept	2.89	0.15	19.88	**<0.001**
Treatment: knockdown	−0.07	0.15	−0.57	0.34
Age: adult	0.52	0.14	3.77	**<0.001**
Age × Treatment	0.61	0.15	4.43	**<0.001**
				
Models	AIC	ΔAIC	Weight	
Model C	36.6	0.0	0.92	
Model A	42.1	5.5	0.06	
Model B	44.4	7.8	0.02	

Shown are the null model (A), the treatment model (B), and a model considering treatment and age (C). Indicated are estimate, SE (std. error), and the respective *t* and *p* values of the intercept and the included fixed factors. To find the best fitting model, the Akaike information criterion (AIC), and the weight of each model were calculated and models were ordered from best to worst fit.

1 bird ID and virus batch are included as random effects in all models. Significant *p* values are marked in bold.

### Song preferences

Overall, females preferred the song of their tutors over unfamiliar song (mean preference for familiar song: 69 ± 16% of all keypecks, see Table 3). Preferences for the familiar tutor song deviated significantly from chance (intercept significantly different from 0) in all treatment and control groups both in CMM-injected (Fig. 5*b*; Table 6) and HVC-injected (Fig. 5*d*; Table 7) females. In HVC there was a significant age × area interaction: adult knockdowns in HVC showed weaker preferences for familiar song than matched controls (*t*_(24)_ = 2.45, *p* = 0.002; Fig. 5*d*) an effect that was absent in females that had received the knockdown in HVC as juveniles.

**Table 6:** GLM results for preference for familiar song across different groups of birds injected into CMM

CMM preference	Estimate	Std. error	*t* value	*p* value
Model A^*1*^ Preference ∼ (1|Bird ID) + (1|Virus batch)				
Intercept	2.71	0.60	4.54	**<0.001**
Model B: Preference ∼ (1|Bird ID) + (1|Virus batch) + Treatment				
Intercept	3.14	1.02	3.07	**0.002**
Treatment: knockdown	−0.74	1.26	−0.59	0.56
Model C: Preference ∼ (1|Bird ID) + (1|Virus batch) + Treatment + Age/Treatment × Age				
Intercept	2.83	1.11	2.55	**0.01**
Treatment: knockdown	−0.74	1.27	−0.59	0.56
Age: adult	0.74	1.27	0.59	0.56
Age × Treatment	0.02	2.55	0.16	0.87
				
Models	AIC	ΔAIC	Weight	
Model A	26.4	0.0	0.62	
Model B	28.1	1.7	0.26	
Model C	29.7	3.3	0.12	

Shown are the null model (A), the Treatment model (B), and a model considering Treatment and Age (C). Indicated are estimate, SE (std. error), and the respective *t* and *p* values of the intercept and the included fixed factors. To find the best fitting model, the Akaike information criterion (AIC), and the weight of each model were calculated and models were ordered from best to worst fit.

1 bird ID and virus batch are included as random effects in all models. Significant *p* values are marked in bold.

**Table 7:** GLM results for preference for familiar song across different groups of birds injected into HVC

HVC preference	Estimate	Std. error	*t* value	*p* value
Model A^*1*^ Preference ∼ (1|Bird ID) + (1|Virus batch)				
Intercept	1.21	0.34	3.5	**<0.001**
Model B: Preference ∼ (1|Bird ID) + (1|Virus batch) + Treatment				
Intercept	1.34	0.50	2.66	**0.008**
Treatment: knockdown	−0.24	0.69	−0.34	0.73
Model C: Preference ∼ (1|Bird ID) + (1|Virus batch) + Treatment + Age/Treatment × Age				
Intercept	1.46	0.62	2.34	**0.02**
Treatment: knockdown	−0.24	0.69	−0.34	0.73
Age: adult	−0.24	0.69	−0.34	0.73
Age × Treatment	1.67	0.43	2.45	**0.002**
				
Models	AIC	ΔAIC	Weight	
Model A	55.7	0.0	0.65	
Model B	57.6	1.9	0.25	
Model C	59.4	3.7	0.10	

Shown are the null model (A), the Treatment model (B) and a model considering Treatment and Age (C). Indicated are estimate, SE (std. error), and the respective *t* and *p* values of the intercept and the included fixed factors. To find the best fitting model, the Akaike information criterion (AIC) and the weight of each model were calculated and models were ordered from best to worst fit.

1 bird ID and virus batch are included as random effects in all models. Significant *p* values are marked in bold.

### Prediction of behavior during preference tests by FoxP1 expression levels

**Figure 6.**
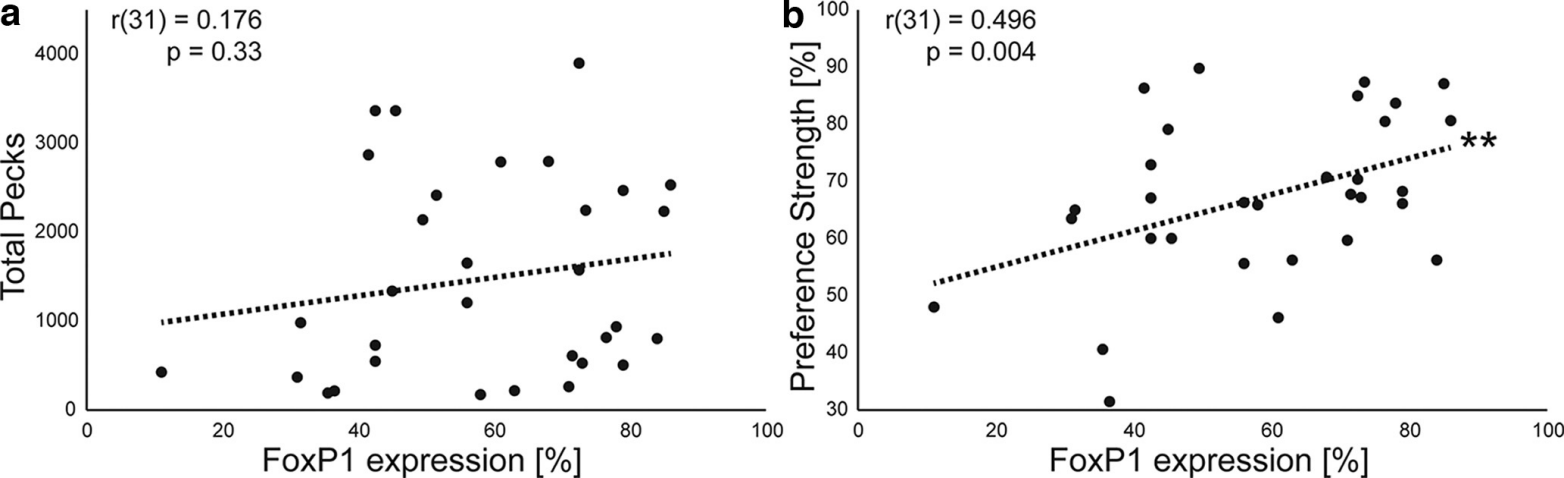
Correlations between relative FoxP1 expression and behavioral measures. Individual points depict relative FoxP1 expression across both hemispheres in relation to a bird’s behavior. Dotted line indicates linear fit of data. ***a***, Relative FoxP1 expression in relation to total number of key pecks during the entire preference test. ***b***, Relative FoxP1 expression in relation to preference strength for familiar song.

As the knockdown efficiency varied and is difficult to compare between individuals because of unknown levels of FoxP1 expression before the injections, we tested whether the FoxP1 expression levels after knockdowns (referred to as knockdown efficiency) could predict keypecking activity and preference strength. The total number of pecks during the preference test did not correlate with knockdown efficiency (Pearson’s *r*(31) = 0.18, *p* > 0.05; Fig. 6*a*). However, the preference strength for familiar song correlated with knockdown efficiency across all groups (Pearson’s *r*(31) = 0.5, *p* = 0.004; Fig. 6*b*). The respective contributions of knockdown efficiency, age group and injected area as well as the interaction of the latter on number of pecks and preference strength were further modelled in GLMs (Tables 8, 9). Total number of pecks by birds with FoxP1 knockdowns during the preference test was predicted by individual FoxP1 expression levels (z(32) = 36.61, *p* < 0.001; Table 8) and age at injection (z(32) = −7.79, *p* < 0.001) as well as area of injection (z(32) = −1.31, *p* < 0.001). The interaction between these latter two factors was also significant (z(32) = 3.17, *p* = 0.002), which further supports behavioral changes occurring only in adult HVC knockdowns (Fig. 5*a–d*). Preference strength for familiar song during the test was also predicted by the relative FoxP1 expression (z(32) = 3.54, *p* < 0.001; Table 9) but was neither affected by injected area nor age during injection.

**Table 8:** GLM results for the total number of pecks as dependent variable in relation to relative FoxP1 expression

Pecking vs FoxP1	Estimate	Std. error	z value	*p* value
Intercept	6.34	0.27	23.72	**<0.001**
Rel. FoxP1 expression	0.02	0.0004	36.61	**<0.001**
Age: juvenile	−1.5	0.02	−7.79	**<0.001**
Area: HVC	−1.31	0.02	−66.2	**<0.001**
Age × Area	1.79	0.57	3.17	**0.002**

Shown are the results of a model including virus batch as random factors, relative FoxP1 expression, injected area, and age during injection and the interaction of age and area as fixed factors as Poisson distributed data. Estimate, SE (std. error), and the respective z and *p* values of the intercept and the included factors are indicated.

Virus batch is included as random effect. Significant *p* values are marked in bold. Full model: Total Pecks ∼ (1|Virus batch) + FoxP1 expression + Age + Area/Age × Area.

**Table 9:** GLM results for the preference for familiar song as dependent variable in relation to relative FoxP1 expression

Preference vs FoxP1	Estimate	Std. error	z value	*p* value
Intercept	3.93	0.11	35.37	**<0.001**
Rel. FoxP1 expression	0.006	0.002	3.56	**<0.001**
Age: juvenile	0.03	0.07	0.49	0.62
Area: HVC	−0.12	0.07	−1.71	0.09

Shown are the results of a model including virus batch as random factors, relative FoxP1 expression, injected area, and age during injection as fixed factors as Poisson distributed data. Estimate, SE (std. error), and the respective z and *p* values of the intercept and the included factors are indicated.

Virus batch is included as random effect. Significant *p* values are marked in bold. Full model: Preference ∼ (1|Virus batch) + FoxP1 expression + Age + Area. Interaction between Age and Area was not significant and excluded from the model.

## Discussion

In this study, we tested for potential functional roles of FoxP1 in the development and maintenance of learned auditory preferences in female zebra finches. *FoxP1* expression was reduced by localized knockdowns in two forebrain areas, CMM and HVC, that are both part of the neural circuit supporting auditory learning. The development of a preference for tutor song was not affected by the reduction of FoxP1 levels reported here: females from all knockdown groups still preferred songs of their tutors over unfamiliar songs as adults. However, the treatment showed area-specific and age-specific effects on the reinforcing quality of (memorized) song: FoxP1 knockdown in adult HVC was associated with a lower motivation to elicit song playback (lower pecking activity) and a weaker preference for familiar song in experimental females versus sham-treated controls. No such differences were observed between treatment groups that had received the knockdown in HVC as juveniles or in CMM (independent of age) and their respective controls. Validation by qPCR analyses confirmed reduced *FoxP1* expression in the target regions in the knockdown groups compared with controls across all treatments. Additionally, preference strength for familiar song was correlated to knockdown efficiency.

Although *FoxP1* expression differed significantly between all controls and knockdowns on a group level, *FoxP1* expression was higher in some knockdown birds compared with matched controls. This might be caused by interindividual variability in endogenous levels of expression of the gene (Chen et al., 2013) as has been shown for *FoxP2* expression (Adam et al., 2017; Kosubek-Langer and Scharff, 2020). Since gene expression can only be measured once per bird it is impossible to know what the birds’ FoxP1 expression levels were before the knockdowns/controls were performed.

Age-specific knockdown effects could have arisen because higher receptor density and synaptic plasticity during development (Ribeiro and Mello, 2000; Wada et al., 2004; Simonyan et al., 2012) may have compensated potential effects of reduced FoxP1 in juvenile but not in adult females. Knockdown buffering is also associated with mRNA decay induced transcription (Haimovich et al., 2013) and possibly increased mRNA turnover in juveniles, nonspecific responses to mRNA manipulation and off-target effects (El-Brolosy et al., 2019). All of these could have contributed to phenotypic variation between the tested groups. However, in light of the large sample size and the use of multiple juvenile and adult control groups, systematic off-target effects are unlikely to explain the observed pattern of results. Accidental targeting of tissue in HVC shelf (an auditory area ventral from HVC; Mello and Clayton, 1994) cannot be excluded, because of the small size of HVC in females. However, endogenous *FoxP1* expression in the HVC shelf area is weak and scattered among only a few cells in comparison to HVC (Extended Data Fig. 2-1*a–a”*; Haesler et al., 2004; Teramitsu et al., 2004; Mendoza et al., 2015), which suggests that potential functional impacts of a knockdown here are less likely than within HVC itself. Furthermore, it was visually validated for each bird that the major part of the injected viral particles transduced cells in HVC. Spillover into HVC shelf cannot be excluded but is not expected to be the predominant functional effect because of lower density of *FoxP1*-expressing cells in this area.

It can be concluded that rather than impairing memory formation the knockdown of *FoxP1* expression in adults seems to affect how auditory input was processed within or relayed from HVC. The observed localized and age-specific effect in HVC aligns with the current understanding of a central role for this brain area in learned recognition and preference in female songbirds. Lesions of HVC in adult females interfere with the behavioral expression of learned song preferences in zebra finches (MacDougall-Shackleton et al., 1998) and canaries (Del Negro et al., 1998; Lehongre and Del Negro, 2011). The prediction that reducing FoxP1 expression in this area in juveniles would affect song preference learning could not be confirmed. In another study, FoxP1 knockdowns at day 35 in HVC of socially raised male zebra finches only impaired song development when the knockdown preceded auditory experiences (Garcia-Oscos et al., 2021). This observation is consistent with the possibility that in male zebra finches, FoxP1 knockdowns in HVC affect the formation of appropriate vocal production memories, different from the present findings of effects on auditory memories in females.

CMM and HVC were tested specifically because both areas are involved in supporting memory, sensory feedback, and motor learning (Bell et al., 2015; Roberts et al., 2017; Soyman and Vicario, 2017; Inda et al., 2020, 2021), and typically show high *FoxP1* expression in juvenile and adult zebra finches (Fig. 2*a*,*g*; Teramitsu et al., 2004; Mendoza et al., 2015). While HVC can be seen as a hub of auditory and motor input, relaying information and input from multiple sources in both the sensory and motor song circuit (Roberts et al., 2012; Lynch et al., 2013), studies of immediate early gene (IEG) expression have implicated CMM in tutor song memory (Bolhuis et al., 2000; Terpstra et al., 2006; Eda-Fujiwara et al., 2016) because neuronal activity increases more after familiar than unfamiliar stimulus presentation. CMM neuronal activity is also associated with auditory perception and discrimination based on extracellular recordings in female (Inda et al., 2020, 2021) and male zebra finches during passive playbacks (Woolley et al., 2005), Go/Nogo tasks (Bell et al., 2015), and functional fMRI (Boumans et al., 2008). In this study, preferences for tutor song were equally strong in CMM knockdowns and untreated control females, suggesting no functional role of FoxP1 in song preference and its acquisition.

Our findings imply a dosage dependent effect of FoxP1 on the reinforcing quality of the tutor song rather than its memorization, which raises the question of how reduced *FoxP1* expression in HVC could have reduced the rewarding qualities of song. FoxP1 knockdowns in mice modify the excitability of medium spiny neurons that express dopamine receptor 1 (Araujo et al., 2015) which is relevant for motivational behaviors (Wise and Rompre, 1989). Most dopamine receptors are highly expressed in HVC of juvenile zebra finches (Kubikova et al., 2010) where blocked dopamine signals impair song copying (Tanaka et al., 2018). Systemic activation of dopamine D2 receptors also affects female song preference (Day et al., 2019b). From our data, it cannot be concluded whether and how FoxP1 influences the motivational system but the regulation of dopamine receptor expression by FoxP1 as a potential candidate mechanism for mediating feedback-based learning and memory is worth further investigation. The results from this study align with the idea of a dopamine driven system supporting rewarding qualities of tutor song perception during development. This system is either sufficiently plastic during development to compensate for reduced FoxP1 expression or it only depends on FoxP1 during maintenance but not during development. Once established, the reward system seems to be (partially) dependent on continuous Foxp1 expression in HVC as the local knockdown in the adults decreased both motivation and preference for hearing the songs that at this age are normally well consolidated and stable (Riebel, 2000, 2003b; Riebel et al., 2009).

From the knockdown studies conducted to date in birds, a picture emerges that implicates several FoxP transcription factors in vocal behaviors and vocal learning. FoxP1 knockdown in the sexually dimorphic Area X of juvenile male zebra finches led to incomplete tutor song copying (Norton et al., 2019). Local FoxP1 knockdowns in HVC of juvenile males suggest that reduced *FoxP1* expression in HVC before animals are exposed to tutors inhibits song learning but if these knockdowns occur after an initial learning period, the birds’ ability to imitate tutor song is not altered (Garcia-Oscos et al., 2021). Knockdowns of FoxP2 in Area X of juvenile male zebra finches altered song structure and learning (Haesler et al., 2007) but these effects were weaker or absent in adult knockdowns where local knockdowns of FoxP2 abolish context dependent song variability but not the overall structure (Murugan et al., 2013; Day et al., 2019a). Notably, overexpression of FoxP2 in Area X also impairs juvenile song learning but alters production of learned song in adults as well (Heston and White, 2015; Day et al., 2019a).

It should be noted that FoxP1 dimerizes with itself and other FoxP transcription factors in regions of overlapping expression (Haesler et al., 2004; Teramitsu et al., 2004; Mendoza et al., 2015), with potential consequences for transcriptional activity and DNA binding (Wang et al., 2003; Li et al., 2004) resulting in different effects during developmental stages. Although FoxP2 and FoxP4 in HVC and CMM are expressed at lower levels than FoxP1 (Haesler et al., 2004; Teramitsu et al., 2004; Mendoza et al., 2015), manipulation of either transcription factor could trigger an imbalance between monomers and dimers and lead to partially overlapping phenotypes (Norton et al., 2019).

For now, combined evidence from expression and knockdown studies and the results presented here support functional involvement of FoxP1 in auditory processing and vocal production learning. Interesting perspectives could arise by comparing animal studies with phenotypic analyses of human *FOXP1* mutations associated with speech and language deficits (Sollis et al., 2016, 2017) but also autism spectrum disorder (ASD) associated variation in sensory feedback processing (Marco et al., 2011). Given that vocal learning progresses in stages and that FoxP1 and FoxP2 have diverse downstream functions (Vernes et al., 2011; Mendoza and Scharff, 2017; Viscardi et al., 2017), both perceptual and productive components of vocal learning could be affected independently or simultaneously.

In conclusion, this study shows that *FoxP1* expression in HVC of adult female zebra finches is involved in motivational behaviors. *FoxP1* expression in HVC might be related to auditory perception and not motor control alone as vocal production requires maintenance or retrieval of auditory information and feedback processing.

## Synthesis

Reviewing Editor: Christine Portfors, Washington State University Decisions are customarily a result of the Reviewing Editor and the peer reviewers coming together and discussing their recommendations until a consensus is reached. When revisions are invited, a fact-based synthesis statement explaining their decision and outlining what is needed to prepare a revision will be listed below. The following reviewer(s) agreed to reveal their identity: NONE.

The reviewers agree that this is an important study as there are few studies that manipulate gene expression in the brain of zebra finches to determine an effect on behavior. This is a novel study because they knockdown FoxP1 in motor and auditory cortical analogues in the female zebra finch. The reviewers agree that the findings are important as they suggest FoxP1 expression in HVC, but not CMM, is tied to determining song preference. The manuscript will be stronger however with more detail providing in the methods and clarification of the results, including the figures.

Main comments

1) Apparent lack of mention of what type of virus was used and other critical details. This is presumed to be lentivirus given the citations in line 138 but for reproducibility these details need to be included. What lentiviral plasmid was used (e.g. FUGW), what promoter was used (neuronal specific vs ubiquitous)? Addgene plasmid numbers if relevant etc. It is presently challenging to interpret the data in the absence of this information.

2) line 68-70. The authors say that FoxP1 is highly expressed in HVC and CMM of both sexes and then cite three papers. It is unclear from these citations that FoxP1 is highly expressed. HVC is clear in males, has it been assessed in adult females? Because lines 68-70 set up the rationale for the entire paper it is important to be specific about expression. Similar comments about NCM and NCL, in line 73. The immunohistochemistry in Fig 2 is unclear on these points. ZEBrA atlas (www.zebrafinchatlas.org) has FoxP1 in males but not females as well.

3) Figure 2: In CMM particularly, it looks as though FoxP1 expression is pan-neuronal or very close to. In part I, the knockdown, there are very few red cells labeled. Should knockdown have only occurred in GFP+ cells? shRNA is encoded in the same virus as the GFP so expression of FoxP1 in GFP- cells should not be affected. Therefore, the mismatch of non-GFP+, FoxP1+ cells shown in this figure is difficult to understand. Is this image (I) representative or an example with few FoxP1 labeled cells? No quantification is reported. The HVC sections look as expected with regards to GFP- FoxP1 expression.

4) Any comments on specificity of HVC injections? HVC in a female is very small compared to males (Nixdorf-Bergweiler 1996). The shelf of HVC, directly adjacent to HVC, is an auditory region. Fig 2 looks to have GFP+ and FoxP1+ cells outside of HVC, presumably in the shelf. If the target nucleus is very small and the adjacent region is involved in the behavior being tested, this is a potential experimental confound. How are the authors sure than inadvertently knocking down FoxP1 in HVC shelf does not affect song preference?

Minor comments

-the use of the word reward in the abstract (line 21). The authors hypothesize in the discussion that the dopaminergic reward system may be involved, which is a valid hypothesis, but unexplored here. Authors should avoid mention of dopaminergic reward outside of the discussion.

-line 342: citation needed for why zRalDH was used as part of validation. No zRalDH shown in figures.

-Fig 2: is there a difference in “red” FoxP1 expression (optical density) in GFP+ cells as compared to non-transduced cells? E.g shown in Fig 2 Garcia-Oscos et al 2021 Science Advances.

-Fig 2 and 5 seem tied to the same theme so it may make more sense for them to be presented back-to-back rather than having peck data in between.

-related to the point about the promoter used, is there any information about what cell types (neuronal vs non-neuronal) FoxP1 is expressed in HVC and CMM? The knockdown validation was from bulk RNA yet the promoter may be targeting non-neuronal cells.

## Author Response

Synthesis Statement for Author (Required):

The reviewers agree that this is an important study as there are few studies that manipulate gene expression in the brain of zebra finches to determine an effect on behavior. This is a novel study because they knockdown FoxP1 in motor and auditory cortical analogues in the female zebra finch. The reviewers agree that the findings are important as they suggest FoxP1 expression in HVC, but not CMM, is tied to determining song preference. The manuscript will be stronger however with more detail providing in the methods and clarification of the results, including the figures.

Main comments

1) Apparent lack of mention of what type of virus was used and other critical details. This is presumed to be lentivirus given the citations in line 138 but for reproducibility these details need to be included. What lentiviral plasmid was used (e.g. FUGW), what promoter was used (neuronal specific vs ubiquitous)? Addgene plasmid numbers if relevant etc. It is presently challenging to interpret the data in the absence of this information.

- We specified the necessary details regarding the viral constructs in line 149ff and provided additional insights regarding the control constructs’ shRNA sequence. A table legend (Table 1) was added as well, resulting in a change of all subsequent Table IDs. The table was also moved to the end of the manuscript with the remaining tables.

2) line 68-70. The authors say that FoxP1 is highly expressed in HVC and CMM of both sexes and then cite three papers. It is unclear from these citations that FoxP1 is highly expressed. HVC is clear in males, has it been assessed in adult females? Because lines 68-70 set up the rationale for the entire paper it is important to be specific about expression. Similar comments about NCM and NCL, in line 73. The immunohistochemistry in Fig 2 is unclear on these points. ZEBrA atlas (www.zebrafinchatlas.org) has FoxP1 in males but not females as well.

- We removed the word ‘highly’ in line 69 to clarify that FoxP1 expression is present in female HVC but not extraordinarily strong compared to e.g. male HVC, while it exceeds expression in the surrounding nidopallium. We further specified that HVC is substantially smaller in females than males, and would also like to note that less research has been conducted on this area in females than in males. Immunohistochemistry and in-situ hybridization in the cited literature show that expression of FoxP1 in the nidopallium is generally low. As overall expression profiles are similar between the sexes, and because we did not notice high expression levels of FoxP1 within the nidopallium of females (see also this study Fig. 2a-b), we think that the focus on mesopallial CMM and HVC is justified due to the elevated levels of FoxP1 compared to the surrounding tissue. Thus, while NCM and NCL may be interesting regions in relation to auditory perception, they do not appear to be relevant targets for a FoxP1 knockdown study. We also added that, for both these areas, FoxP1 expression levels have not been formally evaluated in females before (l77f). From our point of view, the lack of FoxP1 expression patterns in female zebra finches in the ZEBrA atlas does not provide evidence for the absence of FoxP1 in brain areas of females, since female zebra finches are understudied in comparison to males.

3) Figure 2: In CMM particularly, it looks as though FoxP1 expression is pan-neuronal or very close to. In part I, the knockdown, there are very few red cells labeled. Should knockdown have only occurred in GFP+ cells? shRNA is encoded in the same virus as the GFP so expression of FoxP1 in GFP- cells should not be affected. Therefore, the mismatch of non-GFP+, FoxP1+ cells shown in this figure is difficult to understand. Is this image (I) representative or an example with few FoxP1 labeled cells? No quantification is reported. The HVC sections look as expected with regards to GFP- FoxP1 expression.

- We added test results of overall fluorescence within the targeted areas that did not result in significant differences (l429ff). As we pointed out in the manuscript, FoxP1 expression differs between areas and across individuals. As we did not have the capacity to quantify the entirety of HVC or CMM of all tested individuals, we limited our immunohistochemical approach to the validation of the constructs’ presence. Quantification of successful knockdown was originally done using qPCR only, as detection of somatic GFP is difficult in tissue that is only being fixed after slicing occurred, and because most tissue was collected for RNA extraction not allowing for a profound coverage of HVC with immunohistochemical quantification approaches. Due to the high variability of expression across different slices obtained from birds throughout the experiment, the selected images are representative for the entirety of birds. We also now state in the legend of Figure 2 that micrographs were not used to assess the efficiency of the knockdown.

4) Any comments on specificity of HVC injections? HVC in a female is very small compared to males (Nixdorf-Bergweiler 1996). The shelf of HVC, directly adjacent to HVC, is an auditory region. Fig 2 looks to have GFP+ and FoxP1+ cells outside of HVC, presumably in the shelf. If the target nucleus is very small and the adjacent region is involved in the behavior being tested, this is a potential experimental confound. How are the authors sure than inadvertently knocking down FoxP1 in HVC shelf does not affect song preference?

- Figure 2e and k show that both HVC (2e) and CMM (2k) were targeted reliably and in a highly specific manner. Indeed, HVC is much smaller in females than in males, and we further clarified this in l71f, referring to a previous study initially describing size differences of song nuclei (Nottebohm and Arnold, 1976; Nixdorf-Bergweiler, 1996). However, even though we made sure to establish and thoroughly test our adjusted set of coordinates and inject as superficially as possible (also added in l193ff), it cannot be excluded that we also targeted some cells throughout the shelf of HVC, which is indeed an auditory area. In comparison with the surrounding tissue (including shelf) the expression levels of FoxP1 have been shown to be higher within HVC (Haesler et al., 2004; Teramitsu et al., 2004; Mendoza et al., 2015). Furthermore, lesions in HVC have been associated with song preference (MacDougall-Shackleton et al., 1998), which suggests that our results are indeed based on expression differences in HVC itself. Still, we agree with the reviewer that we cannot fully exclude a contribution of shelf and so have rephrased the previously existing statements regarding the involvement of shelf in the discussion of the manuscript (l556ff).

Minor comments

-the use of the word reward in the abstract (line 21). The authors hypothesize in the discussion that the dopaminergic reward system may be involved, which is a valid hypothesis, but unexplored here. Authors should avoid mention of dopaminergic reward outside of the discussion.

- We rephrased the last sentence to avoid the use of the word “reward”. The last sentence of the abstract now reads in l21ff: “In summary, FoxP1 knockdowns in the cortical song nucleus HVC were not associated with impaired tutor song memory but reduced motivation to actively request tutor songs.”

-line 342: citation needed for why zRalDH was used as part of validation. No zRalDH shown in figures.

- A citation to Roeske et al. 2014 has been added in line 355ff. zRalDH is now shown in the inlet a’ of Figure 2.

-Fig 2: is there a difference in “red” FoxP1 expression (optical density) in GFP+ cells as compared to non-transduced cells? E.g shown in Fig 2 Garcia-Oscos et al 2021 Science Advances.

- We did not test this on a large scale due to the lack of sufficient slides to provide an overview of the entire HVC of the females tested during our study. The majority of tissue from all birds was used for RNA extraction and further RNA processing that is part of another study. Furthermore, as brain slices were produced from unfixed tissue, immunohistochemical analyses are expected to be less reliable than gene expression analyses with qPCR which we used to determine the efficiency of the knockdowns. However, based on images from a subset of birds, we quantified GFP/FoxP1 coexpression in the targeted areas similar to Garcia-Oscos et al. 2021 (line 432ff).

-Fig 2 and 5 seem tied to the same theme so it may make more sense for them to be presented back-to-back rather than having peck data in between.

- We agree, that showing former Figures 2 (microscopy) and 5 (qPCR results) in a back-to-back fashion helps to present the data in a more coherent way and have thus moved Figure 2 into the results section right before former Figure 5 which is now Figure 3. Along this adjustment, the text refers to Figure 2 now from l429ff and we moved three sentences describing potential explanations for expression differences into the discussion (l540ff). Figure 3 is referred to from line 440ff.

-related to the point about the promoter used, is there any information about what cell types (neuronal vs non-neuronal) FoxP1 is expressed in HVC and CMM? The knockdown validation was from bulk RNA yet the promoter may be targeting non-neuronal cells.

- To further clarify the cell types that express FoxP1, we added more detail in the introduction lines 79ff. While it is true that the promoter may target non-neuronal cells, the short hairpin constructs that we used are specifically designed against FoxP1 which is only neuronally expressed. Thus, these should not have an effect beyond unspecific responses towards short-hairpins, which we would also expect to observe in our control constructs.

-Additional changes:

- While working on the revisions we noticed that for clarity the table legends of tables 3 and 4 could be streamlined to match the legends of tables 5 and 6. Note that only the wording, not the content has been changed.

The original description was as follows “Shown are the results of model A including only bird ID and virus batch as random factors, model B which added treatment as a fixed factor, and a model adding treatment and age as fixed factors (model C). For all models, estimate, standard error (std. error) and the respective t and p values of the intercept and the included fixed factors are indicated. To find the best fitting model, the Akaike information criterion (AIC) and the weight of each model were calculated and models were ordered from best to worst fit.”

This has been adjusted to the following wording: “Shown are the null model (A), the Treatment model (B) and a model considering Treatment and Age (C). Indicated are estimate, standard error (std. error) and the respective t and p values of the intercept and the included fixed factors. To find the best fitting model, the Akaike information criterion (AIC) and the weight of each model were calculated and models were ordered from best to worst fit.”
