# Supplementary material for: Effects of Cortical FoxP1 Knockdowns on Learned Song Preference in Female Zebra Finches
Source: eNeuro. 2023 Mar 28;10(3):ENEURO.0328-22.2023. doi: 10.1523/ENEURO.0328-22.2023 (PMC10062489; doi:10.1523/ENEURO.0328-22.2023)
Supplement: Extended Data Figure 1-1 — Test stimuli during preference tests of all birds. Bird IDs with an asterisk indicate playbacks that were used for multiple stimulus pairings. Download Figure 1-1, DOC file. [file enu-eN-NWR-0328-22-s02.doc]

**Figure 1-1**:

| Bird ID | Pair | # Elements | # Motifs | Total duration stimulus song [s] | Difference duration stimulus set [%] |
| --- | --- | --- | --- | --- | --- |
| 3089 | 1 | 5 | 6 | 5.56 | 1.83 |
| g13r8* | 1 | 5 | 6 | 5.46 |
| 3740 | 2 | 6 | 7 | 5.59 | 2.19 |
| p10r8 | 2 | 5 | 7 | 5.47 |
| 4236* | 3 | 6 | 5 | 5.87 | 0.17 |
| 4389* | 3 | 5 | 5 | 5.88 |
| 4396 | 4 | 4 | 7 | 5.5 | 0.36 |
| 4575 | 4 | 4 | 7 | 5.48 |
| 4786* | 5 | 4 | 5 | 6.8 | 1.02 |
| 5156* | 5 | 5 | 5 | 6.87 |
| 2137 | 6 | 5 | 5 | 5.83 | 0.68 |
| 4236* | 6 | 6 | 5 | 5.87 |
| 2804 | 7 | 5 | 5 | 5.85 | 1.39 |
| 4512 | 7 | 6 | 5 | 5.77 |
| 4748 | 8 | 5 | 6 | 5.55 | 1.65 |
| g13r8* | 8 | 5 | 6 | 5.46 |
| 5013 | 9 | 4 | 6 | 5.32 | 0.37 |
| 5141 | 9 | 4 | 6 | 5.34 |
| 3653 | 10 | 5 | *7* | 5.16 | 0 |
| 5679 | 10 | 5 | *5* | 5.16 |
| 5492* | 11 | 4 | 5 | 5.29 | 5.2 |
| 4532 | 11 | 5 | 5 | 5.58 |
| 5832 | 12 | 4 | 5 | 5.47 | 0.92 |
| 4217 | 12 | 5 | 5 | 5.42 |
| 4479 | 13 | 4 | 5 | 5.48 | 0.9 |
| 5650 | 13 | 4 | 5 | 5.53 |
| 4786* | 14 | 4 | 5 | 6.8 | 1.02 |
| 5156* | 14 | 5 | 5 | 6.87 |
| 4170 | 15 | 5 | 5 | 5.59 | 4.93 |
| 4389* | 15 | 5 | 5 | 5.88 |
| 5788 | 16 | 5 | 3 | 5.04 | 0.8 |
| 5492* | 16 | 4 | 4 | 5.00 |
| 5804 | 17 | 5 | 5 | 6.46 | 1.41 |
| 4042 | 17 | 5 | 5 | 6.37 |
