# Supplementary material for: Effects of Cortical FoxP1 Knockdowns on Learned Song Preference in Female Zebra Finches
Source: eNeuro. 2023 Mar 28;10(3):ENEURO.0328-22.2023. doi: 10.1523/ENEURO.0328-22.2023 (PMC10062489; doi:10.1523/ENEURO.0328-22.2023)
Supplement: Extended Data Table 1-1 — Viral constructs which were used during this study resulting in different virus batches distributed among the experimental groups. Indicated are the experimental condition they belong to, either control or knockdown, the serotype of one control construct and two knockdown versions of a short-hairpin construct, their respective production date and area, age group, and number of birds they were injected into. Download Table 1-1, DOC file. [file enu-eN-NWR-0328-22-s05.doc]

**Table 1-1**

| Experimental group | Serotype | Production date | Region | Age | Birds injected |
| --- | --- | --- | --- | --- | --- |
| Control | shCTRL | 19.02.2016 | CMM | Adult | 2 |
| Control | shCTRL | 19.02.2016 | CMM | Adult | 1 |
| Control | shCTRL | 03.02.2017 | CMM | Adult | 4 |
| Control | shCTRL | 21.07.2017 | CMM | Adult | 6 |
| Control | shCTRL | 21.07.2017 | CMM | Juvenile | 12 |
| Control | shCTRL | 10.10.2014 | HVC | Adult | 11 |
| Control | shCTRL | 03.02.2017 | HVC | Adult | 1 |
| Control | shCTRL | 13.01.2017 | HVC | Juvenile | 5 |
| Control | shCTRL | 03.02.2017 | HVC | Juvenile | 5 |
| Control | shCTRL | 21.07.2017 | HVC | Juvenile | 2 |
| Knockdown | shKRAK | 10.10.2014 | CMM | Adult | 1 |
| Knockdown | shKRAK | 03.02.2017 | CMM | Adult | 2 |
| Knockdown | shKRAK | 21.07.2017 | CMM | Adult | 2 |
| Knockdown | shKRAK | 21.07.2017 | CMM | Juvenile | 6 |
| Knockdown | shKRAK | 10.10.2014 | HVC | Adult | 6 |
| Knockdown | shKRAK | 03.02.2017 | HVC | Adult | 1 |
| Knockdown | shKRAK | 13.01.2017 | HVC | Juvenile | 6 |
| Knockdown | shY31 | 10.10.2014 | CMM | Adult | 2 |
| Knockdown | shY31 | 19.02.2016 | CMM | Adult | 1 |
| Knockdown | shY31 | 03.02.2017 | CMM | Adult | 1 |
| Knockdown | shY31 | 21.07.2017 | CMM | Adult | 3 |
| Knockdown | shY31 | 21.07.2017 | CMM | Juvenile | 6 |
| Knockdown | shY31 | 10.10.2014 | HVC | Adult | 3 |
| Knockdown | shY31 | 03.02.2017 | HVC | Adult | 1 |
| Knockdown | shY31 | 21.07.2017 | HVC | Adult | 1 |
| Knockdown | shY31 | 19.02.2016 | HVC | Juvenile | 1 |
| Knockdown | shY31 | 13.01.2017 | HVC | Juvenile | 5 |
