# Supplementary material for: Effects of Cortical FoxP1 Knockdowns on Learned Song Preference in Female Zebra Finches
Source: eNeuro. 2023 Mar 28;10(3):ENEURO.0328-22.2023. doi: 10.1523/ENEURO.0328-22.2023 (PMC10062489; doi:10.1523/ENEURO.0328-22.2023)
Supplement: Extended Data Figure 3-1 — Primers used for qPCR validation of knockdown in RNA extracted from zebra finch target regions HVC and CMM. Download Figure 3-1, DOC file. [file enu-eN-NWR-0328-22-s04.doc]

**Figure 3-1**

| Gene | Ensembl ID | Forward sequence | Reverse sequence | Amplicon Length |
| --- | --- | --- | --- | --- |
| HMBS | ENSTGUG00000000010 | 5’-GCAGCATGTT  GGCATCACAG-3’ | 5’-TGCTTTGCTC  CCTTGCTCAG-3’ | 88 bp |
| FoxP1 | ENSTGUG00000009872 | 5’-CGTTAAAGGG  GCAGTATGGA-3’ | 5’-GCCATTGAAG  CCTGTAAAGC-3’ | 130 bp |
