# Supplementary figures and images for: Effects of Cortical FoxP1 Knockdowns on Learned Song Preference in Female Zebra Finches
Source: eNeuro. 2023 Mar 28;10(3):ENEURO.0328-22.2023. doi: 10.1523/ENEURO.0328-22.2023 (PMC10062489; doi:10.1523/ENEURO.0328-22.2023)

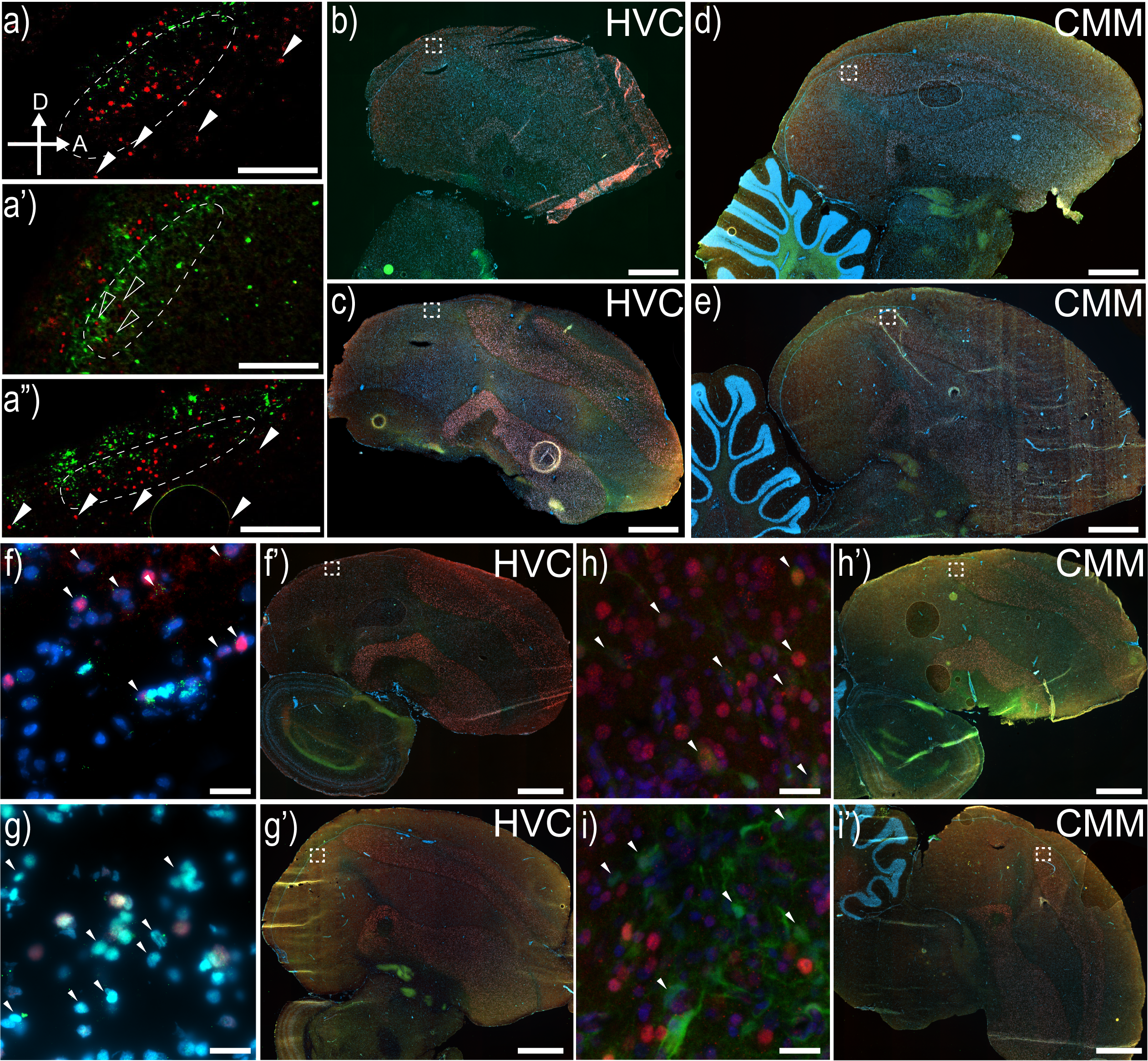

Supplement: Extended Data Figure 2-1 — Immunohistochemistry of HVC or CMM-injected birds after completion of the experiments. Shown are merged stainings of GFP (green) indicating virus-infected neurons, and FoxP1 immunoreactivity (red). a–a”, Sagittal images from three different females highlighting the difference of FoxP1-expressing cells in HVC (dashed line). Filled arrowheads in a and a” point towards FoxP1-positive cells ventral from HVC that might be located in the HVC-shelf. Empty arrowheads in a’ show FoxP1-expressing neurons in HVC. b–e, Sagittal scans of the corresponding high magnification images (dashed squares) of females injected as adults shown in Figure 2b,c,h,i, respectively. b, d, Slices from females injected with control constructs. c, e, Slices from females injected with knockdown constructs, respectively. f, Close up of HVC from a bird injected into HVC with the control construct as juvenile. f’, Sagittal overview of the brain slice corresponding to f. Dashed outline highlights the coordinates of f. Arrowheads indicate cells expressing both GFP and FoxP1. g, Close up of HVC from a bird injected into HVC with a knockdown construct as juvenile. g’, Sagittal overview of the brain slice corresponding to g. Dashed outline highlights the coordinates of g. Arrowheads indicate cells expressing GFP but not FoxP1. h, Close up of CMM from a bird injected into CMM with the control construct as juvenile. h’, Sagittal overview of the brain slice corresponding to h. Dashed outline highlights the coordinates of h. Arrowheads indicate cells expressing both GFP and FoxP1. i, Close up of CMM from a bird injected into CMM with a knockdown construct as juvenile. i’, Sagittal overview of the brain slice corresponding to i. Dashed outline highlights the coordinates of i. Arrowheads indicate cells expressing GFP but not FoxP1. Scale bars: 200 μm (a–a”), 1000 μm (b–e, f’, g’, h’, i’), and 20 μm (f, g, h, i). Download Figure 2-1, TIF file. [file enu-eN-NWR-0328-22-s03.tif]
